# A mathematical model for IL-6-mediated, stem cell driven tumor growth and targeted treatment

**DOI:** 10.1371/journal.pcbi.1005920

**Published:** 2018-01-19

**Authors:** Fereshteh Nazari, Alexander T. Pearson, Jacques Eduardo Nör, Trachette L. Jackson

**Affiliations:** 1 Simon A. Levin Mathematical, Computational, and Modeling Sciences Center, School of Human Evolution and Social Change, Arizona State University, Tempe, Arizona, United States of America; 2 Division of Hematology/Oncology, Department of Internal Medicine, University of Michigan Cancer Center, Ann Arbor, Michigan, United States of America; 3 Departments of Cardiology, Restorative Sciences, and Endontics, University of Michigan, Ann Arbor, Michigan, United States of America; 4 Department of Mathematics, University of Michigan, Ann Arbor, Michigan, United States of America; Oxford, UNITED KINGDOM

## Abstract

Targeting key regulators of the cancer stem cell phenotype to overcome their critical influence on tumor growth is a promising new strategy for cancer treatment. Here we present a modeling framework that operates at both the cellular and molecular levels, for investigating IL-6 mediated, cancer stem cell driven tumor growth and targeted treatment with anti-IL6 antibodies. Our immediate goal is to quantify the influence of IL-6 on cancer stem cell self-renewal and survival, and to characterize the subsequent impact on tumor growth dynamics. By including the molecular details of IL-6 binding, we are able to quantify the temporal changes in fractional occupancies of bound receptors and their influence on tumor volume. There is a strong correlation between the model output and experimental data for primary tumor xenografts. We also used the model to predict tumor response to administration of the humanized IL-6R monoclonal antibody, tocilizumab (TCZ), and we found that as little as 1mg/kg of TCZ administered weekly for 7 weeks is sufficient to result in tumor reduction and a sustained deceleration of tumor growth.

## Author summary

A small population of cancer stem cells that share many of the biological characteristics of normal adult stem cells are believed to initiate and sustain tumor growth for a wide variety of malignancies. Growth and survival of these cancer stem cells is highly influenced by tumor micro-environmental factors and molecular signaling initiated by cytokines and growth factors. This work focuses on quantifying the influence of IL-6, a pleiotropic cytokine secreted by a variety of cell types, on cancer stem cell self-renewal and survival. We present a mathematical model for IL-6 mediated, cancer stem cell driven tumor growth that operates at the following levels: (1) the molecular level—capturing cell surface dynamics of receptor-ligand binding and receptor activation that lead to intra-cellular signal transduction cascades; and (2) the cellular level—describing tumor growth, cellular composition, and response to treatments targeted against IL-6.

## Introduction

It is widely believed, based on increasing evidence, that a small population of tumorigenic cells, which are in many ways similar to normal adult stem cells, is responsible for the initiation and maintenance of malignant tumors [1–5]. This concept, termed the cancer stem cell (CSC) hypothesis, takes the view that tumors, like adult tissues, arise from multipotent cells that exhibit the ability to self-renew as well as give rise to differentiated tissue cells [4–7]. It is hypothesized that CSCs are responsible for tumor initiation, progression, resistance and recurrence [4, 6, 8]. Cancer stem cells have now been identified in a variety of malignancies, including tumors of the blood, breast, colon, brain, and head and neck [8].

Head and neck squamous cell carcinoma (HNSCC), a highly invasive form of cancer, is the sixth most common cancer in the world, with over 600,000 new cases diagnosed globally each year [9]. The identification of cancer stem cells as *drivers* of the tumorigenic process in HNSCC [4] provides a rationale for the targeted elimination of these cells in HNSCC tumors. It is well known that growth and survival of CSCs is highly influenced by tumor micro-environmental factors and molecular signaling, initiated by cytokines and growth factors [10–13]. IL-6 is a pleiotropic cytokine, secreted by a variety of cell types, that is a key player in number of cellular processes including proliferation, survival, differentiation, migration and invasion [14]. It is also commonly overexpressed in most cancer types including HNSCC [8, 14, 15].

High IL-6 expression independently predicts tumor recurrence, tumor metastasis and poor survival in head and neck cancer patients [14]. IL-6 signaling is mediated by binding to its natural receptor, IL-6R and the universally expressed gp130 receptor. Once bound to IL6, the IL-6R-gp130 complex results in the phosphorylation of STAT3, which is indicative of stemness [8]. Recent evidence shows that IL-6R is overexpressed on CSCs and IL-6 secreted by both tumor cells and endothelial cells (ECs) enhances the survival, self-renewal and tumor initiation potential of cancer stem cells in HNSCC [8]. Given that HNSCC has a 5-year survival rate of less than 60%, which has improved little over the last 20 years [16], these studies of the impact of IL-6 on CSCs provide strong motivation for the development of anti-IL-6 therapies for the targeted treatment of HNSCC.

The fact that CSCs form only a small portion of the total tumor burden, but may play a disproportionately important role in determining tumor growth and treatment outcomes makes them an important cellular phenotype in need of further study. In this paper, we develop a predictive computational framework that aims to advance our current understanding of the differential impact of IL-6 on CSC self-renewal and HNSCC growth and investigate the mechanisms of tumor reduction associated targeted treatment with the anti-IL-6R antibody Tocilizumab.

While numerous models of cancer stem cell driven tumor growth exist (see [17] for a review), connecting mathematical models of the CSC hypothesis to experimental data, either at the molecular and cellular scale or at the clinical scale is far less common, for examples see [18–21]. Our model is unique in that it includes the molecular level details IL-6 signal initiation and its effect on tumor cell survival and proliferation, while also capturing the influence of IL-6 on the probability of self-renewal for cancer stem cells. To our knowledge, this is the first model of cancer stem cell driven tumor growth that operates across molecular and cellular scales. Our model allows for the quantification of the temporal changes fractional occupancies of bound IL-6 receptors and their impact on tumor growth dynamics, which is precisely the level of detail required to better understand targeted therapies that antagonize IL-6 signaling.

## Materials and methods

Below we develop a mathematical model for cancer stem cell-driven tumor growth that is designed to quantify the influence of tumor secreted IL-6 signaling on cancer growth and tissue composition. We extend the model to include treatment with the anti-IL-6R antibody, Tocilizumab (TCZ).

### Modeling cancer stem cell driven tumor growth pre-treatment

**Fig 1:**
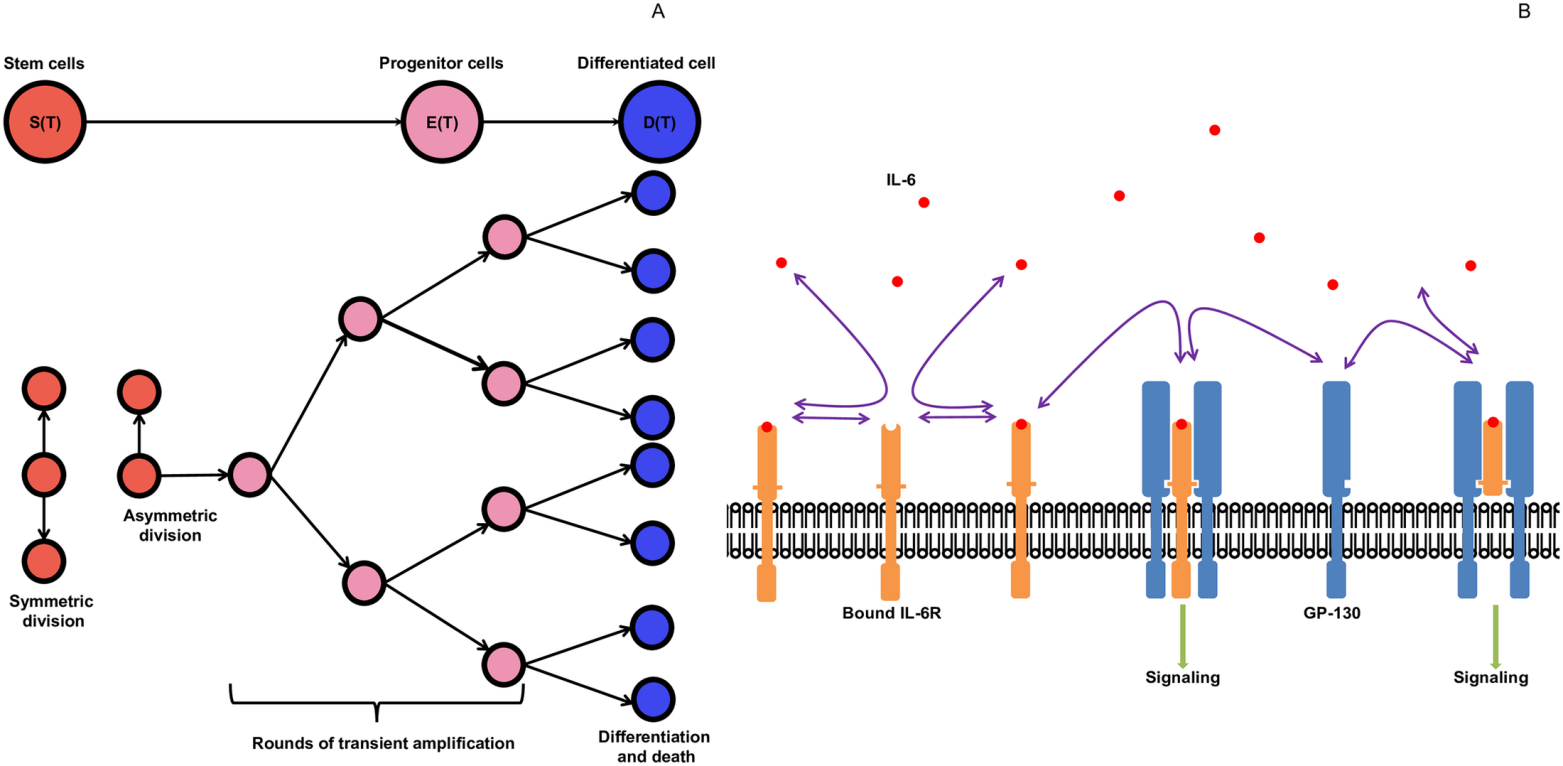
Schematic diagram of the cellular and molecular processes considered in the mathematical model. A: stem cell division (this figure was adapted from Fig. 1 in [24] B: IL-6 binding dynamics.

This mathematical model has as its foundation specific biological knowledge of the function of IL-6 signaling and the differential cellular responses to it. The pre-treatment model tracks the temporal evolution of three cancer cell types (stem, progenitor, and terminally differentiated) as well as IL-6 and membrane bound IL-6 receptors (IL-6R) in their free and bound forms as depicted in Fig 1. Although a soluble form of IL-6R (sIL-6R) exists and can bind IL-6 with a similar affinity as the membrane bound form [22], we choose to simplify our modeling approach by not including sIL-6R because there is evidence that its role is most important during *trans* signaling when cells lack membrane bound IL-6R [14, 22, 23].

Table 1 lists each model variable along with its units.

**Table 1:** List of model variables.

Variable	Description	Units
*S*	HNSCC stem cells	# of cells
*E*	HNSCC progenitor tumor cells	# of cells
*D*	HNSCC differentiated tumor cells	# of cells
*L*	IL-6	fmol
*R*_*S*_	IL-6R on stem cells	fmol
*C*_*S*_	IL-6, cell bound IL-6R complex on stem cells	fmol
*R*_*E*_	IL-6R on progenitor cells	fmol
*C*_*E*_	IL-6, cell bound IL-6R complex on progenitor cells	fmol
*R*_*D*_	IL-6R on differentiated cells	fmol
*C*_*D*_	IL-6, cell bound IL-6R complex on differentiated cells	fmol

#### Cancer cell equations

The total cellular composition of an HNSCC tumor at time *t* is divided into cancer stem cells *S*(*t*), transient amplifying/progenitor cancer cells *E*(*t*) and terminally differentiated cancer cells *D*(*t*) (see Fig 1A). Tumor cell secreted-IL-6, denoted by *L*(*t*), binds to unoccupied (free) IL-6 receptors (IL-6R) on the surface of stem, progenitor and differentiated cells in amounts denoted by *R*_*S*_, *R*_*E*_, and *R*_*D*_; respectively. Association of IL-6 to IL-6R results in the formation of IL-6/IL-6R complexes, whose quantities are represented by *C*_*S*_(*t*), *C*_*E*_(*t*) and *C*_*D*_(*t*), respectively, for each cell type.

Eq (1), below, describes temporal changes in the cancer stem cell (CSC) population:

$$\underset{\text{Stem}\;\text{cell}}{\underset{︸}{\frac{dS}{dt}}}=\underset{\text{Stem}\;\;\text{cell}\;\;\text{self-renewal}}{\underset{︸}{\alpha_{S}P_{S}\left(S,\phi_{S}\right)S}}-\underset{\text{Stem}\;\;\text{cell}\;\;\text{death}}{\underset{︸}{\frac{\delta_{S}}{1+\gamma_{S}\phi_{S}}S\;}}.$$ (1)

CSCs divide at rate, *α*_*S*_. The first term in (1) assumes that CSCs can either symmetrically renew, creating two identical daughter cells that retain *stemness*, or they can asymmetrically differentiate into one stem and one progenitor cell. The self-renewal probability, *P*_*S*_(*S*, *ϕ*_*S*_), varies depending on the total cancer stem cell population size and on the fractional occupancy of bound IL-6R per cell defined below in (2):

$$\begin{array}{c} \phi_{S}=\frac{1}{R_{T_{S}}}\;\frac{C_{S}}{S}, \end{array}$$ (2)

where $R_{T_{S}}$ is the total number of IL-6 receptors per stem cell.

The second term of (1) assumes that CSCs die with a maximum death rate, *δ*_*S*_. There is evidence that IL-6 enhances the survival of cancer stem-like cells [8], therefore this term also describes the decrease in cell death as the fractional occupancy of bound receptors per cell, *ϕ*_*S*_, increases.

Eqs (3) and (4) describe temporal changes in the progenitor and terminally differentiated cell populations.

$$\frac{dE}{dt}=\underset{\begin{array}{c} \text{Amplified}\\ \text{stem}\;\text{cell}\;\text{differentiation} \end{array}}{\underset{︸}{A_{in}\alpha_{S}(1-P_{S}(S,\phi_{S}))S}}-\underset{\begin{array}{c} \text{Progenitor}\;\text{cell}\\ \;\text{differentiation} \end{array}}{\underset{︸}{\alpha_{E}E}}-\underset{\text{progenitor}\;\text{cell}\;\text{death}}{\underset{︸}{\frac{\delta_{E}}{1+\gamma_{E}\phi_{E}}E,}}$$ (3)

$$\frac{dD}{dt}=\underset{\begin{array}{c} \text{progenitor}\;\text{cell}\\ \text{differentiation} \end{array}}{\underset{︸}{A_{out}\alpha_{E}E}}-\underset{\text{differentiated}\;\text{cell}\;\text{death}}{\underset{︸}{\frac{\delta_{D}}{1+\gamma_{D}\phi_{D}}D}}$$ (4)

Progenitor cells (3) undergo a limited number (*w*) of mitotic cycles, so called transit-amplifying (TA) cell divisions, before entering a post-mitotic terminally differentiated state [1, 25]. In this model, instead of adding *w* sub-compartments of progenitor cells, it is assumed that each stem cell is amplified upon entry into the progenitor pool. This is a simplified version of the model developed in [26] and this approach has also been used in [27]. These assumptions concerning amplification imply that the efflux from the stem compartment is augmented by a factor *A*_*in*_ as soon as the cells enter the progenitor pool as shown in the first term in (3).

The second term in (3) assumes that progenitor cells transition to fully differentiated cells via cell division at a rate *α*_*E*_. Similar to stem cells, progenitor cells die with maximum death rate *δ*_*E*_, which decreases as the fractional occupancy of bound receptors per cell, $\phi_{E}=\frac{1}{R_{T_{E}}}\;\frac{C_{E}}{E}$, increases, where $R_{T_{E}}$ is the total concentrations of receptors per cell.

Immediately before leaving the progenitor compartment, cells are further amplified by a factor *A*_*out*_ = 2 because the transition from progenitor to terminally differentiated cells results in the loss of one progenitor cell and the gain of two terminally differentially cells as shown in the first term in (4). These two amplification factors are selected such that *A*_*in*_ × *A*_*out*_ = 2^*w*^, where *w* = the number of successive stages of TA cell divisions before transforming into mature cell [26]. Terminally differentiated cells live for a specified amount of time and then die at a rate *δ*_*D*_. Similar to progenitor cells, the death rate of terminally differentiated cells decreases as the fractional occupancies of bound receptors per cell, $\phi_{D}=\frac{1}{R_{T_{D}}}\;\frac{C_{D}}{D}$, increases, where $R_{T_{D}}$ is the total concentrations of receptors per cell.

#### The probability of cancer stem cell self-renewal

In the absence of tumor cell-secreted IL-6, the probability of CSC self-renewal, *P*_*S*_, can be regulated by extrinsic and intrinsic chemical signaling as well as environmental (niche) constraints [10–13]. CSC niches are specialized and anatomically distinct microenvironments within the tumor that regulate CSC fate [28]. CSC niches provide signals in the form of both cell-to-cell contacts and secreted factors that stimulate CSC self-renewal, and other stemness characteristics of them [28–30]. Therefore, it is necessary for CSCs to interact with their niche to preserve their *stemness* and ability to self-renewal. However, the physically limited size of the cancer niches [31] may impact the availability of the interaction sites and necessary chemical signals. That is, as the number of CSCs increases, the spatial access of the new born daughter cells to their niche cues becomes more and more limited.

Certain environmental cues can promote self-renewal, while others promote differentiation. Similarly, proteins produced by stem cells themselves can affect self-renewal in an autocrine manner [32, 33]. There is also biological evidence that stem cell regulation in niches may support differentiation and suppress self-renewal. Namely, [34] reports that the niche may support CSC proliferation and differentiation rather than stimulating CSC self-renewal over time [34]. Taken together, all of these reported findings regarding the stem cell niche suggest that the probability of CSC self-renewal is a monotonic (deceasing) function of CSCs and our functional form below reflects this.

Many published mathematical models use a Hill function [27, 35–37], which can be derived from receptor-ligand binding kinetics, to describe the effect of chemical signals on the probability of symmetric self-renewal. For these reasons, our model assumes the following functional form for the probability of stem cell-self renewal in the absence of IL-6:

$$\begin{array}{c} P_{S}\left(S\right)=\frac{\left(P_{S_{max}}-P_{S_{min}}^{*}\right)P_{N_{s}}^{n}}{P_{N_{s}}^{n}+S^{n}}+P_{S_{min}}^{*}\cdot \end{array}$$ (5)

**Fig 2:**
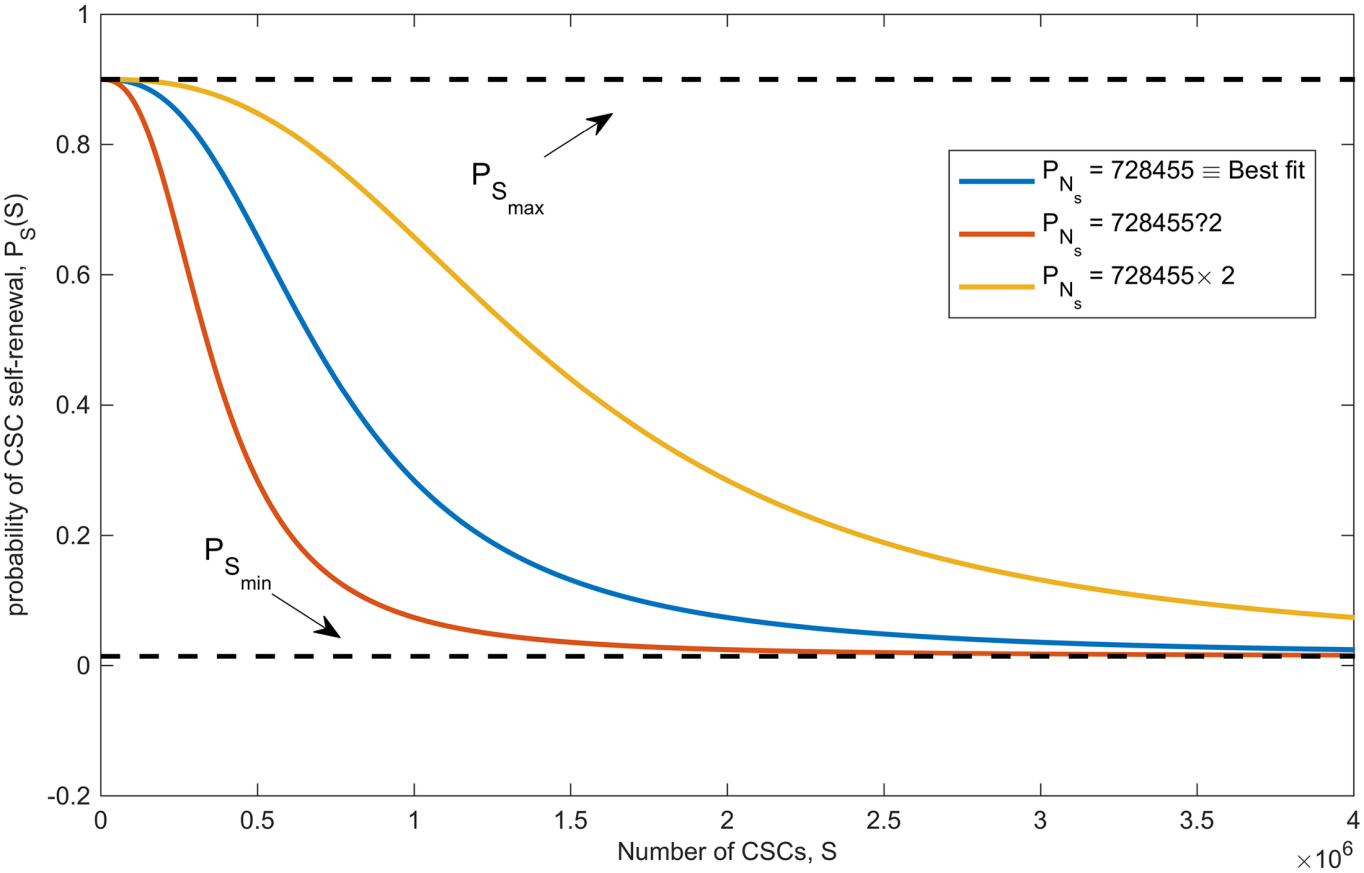
The probability of stem cell self-renewal, *P*_*S*_, as a function of stem cell number.

As the number of cancer stem cells approaches zero, the probability of symmetric self-renewal approaches the maximum value, $P_{S_{max}}$. Conversely, as the number of CSCs approaches infinity, the probability of symmetric self-renewal approaches a minimum value, $P_{S_{min}}^{*}$. The parameter $P_{N_{s}}$ may be interpreted as the number of stem cells for which the probability of symmetric self-renewal is halfway between the maximum and minimum values. Higher values of the exponent *n* > 1 increase the sensitivity of stem cells to signals that promote symmetric self-renewal. Fig 2 plots the probability of CSC self-renewal as a function of cell number for the baseline parameters of the model and for two different choices of the parameter $P_{N_{s}}$.

There is experimental evidence supporting the fact that IL-6 impacts cancer stem cell self-renewal and that the proportion of CSCs increases due to the presence of IL-6 [8]. Therefore, in the presence of tumor cell-secreted IL-6, we modify the functional form of *P*_*S*_ given by (5) so that it still decreases as *S* increases, but also increases as the fraction of bound receptors per cell $\phi_{S}=\frac{C_{S}}{R_{T_{S}}S}$ increases. There are many assumptions we could make for these modifications. For now we assume that $P_{S_{max}}$ remains unchanged (and constant), but that the minimum probability for self-renewal when IL-6 is present, $P_{S_{min}}\left(\phi_{S}\right)$, increases as the amount of bound IL-6 receptors increases. This helps to ensure that IL-6 will impact stem cell fate when the tumor is large. Together, these assumptions yield:

$$\begin{array}{cc} P_{S}\left(S,\phi_{S}\right) & =\frac{\left(P_{S_{max}}-P_{S_{min}}\left(\phi_{S}\right)\right)P_{N_{s}}^{n}}{P_{N_{s}}^{n}+S^{n}}+P_{S_{min}}\left(\phi_{S}\right),\\ P_{S_{min}}\left(\phi_{S}\right) & =\mu_{S}(P_{S_{max}}-P_{S_{min}}^{*})\phi_{S}+P_{S_{min}}^{*} \end{array}$$ (6)

In Eq (6), $P_{S_{min}}^{*}$ is the minimum probability of CSC self-renewal when there is no IL-6 present in the tumor (i.e. $P_{S_{min}}\left(0\right)=P_{S_{min}}^{*}$), and *μ*_*S*_ < 1 is a modulation parameter that adjusts the effects of IL-6 (via *ϕ*_*S*_) on the probability of CSC self-renewal. The equation for $P_{S_{min}}\left(\phi_{S}\right)$ ensures that when IL-6 is present, the minimum probability for self-renewal will increase with *ϕ*_*S*_ to at most $\mu_{S}\left(P_{S_{max}}-P_{S_{min}}^{*}\right)+P_{S_{min}}^{*}<P_{S_{max}}$.

### IL-6-related equations

Eq (7) describes the association, at rate *k*_*f*_, and dissociation, at rate *k*_*r*_, of IL-6 (*L*) to its cell-bound receptors (*R*_*S*_, *R*_*E*_ and *R*_*D*_) on stem, progenitor and differentiated cells; respectively (Fig 1B). An underlying assumption in this equation is that the binding rates are the same, independent of cell type. IL-6 is removed via natural decay at rate λ_*L*_ and is produced by tumor cells at rate *ρ*.

$$\begin{array}{ll} \frac{dL}{dt}=\; & -\underset{\begin{array}{c} \text{IL}-6\;\text{binding}\;\text{to}\\ \text{stem}\;\text{cells} \end{array}}{\underset{︸}{k_{f}LR_{S}}}\;+\;\underset{\begin{array}{c} \text{IL}-6\;\text{dissociation}\\ \text{from}\;\text{stem}\;\text{cells} \end{array}}{\underset{︸}{k_{r}C_{S}}}\;-\;\underset{\begin{array}{c} \text{IL}-6\;\text{binding}\;\text{to}\\ \text{progenitor}\;\text{cells} \end{array}}{\underset{︸}{k_{f}LR_{E}}}\;+\;\underset{\begin{array}{c} \text{IL}-6\;\text{dissociation}\\ \text{from}\;\text{progenitor}\;\text{cells} \end{array}}{\underset{︸}{k_{r}C_{E}}}\\ & -\underset{\begin{array}{c} \text{IL}-6\;\text{binding}\;\text{to}\\ \text{differentiated}\;\text{cells} \end{array}}{\underset{︸}{k_{f}LR_{D}}}\;+\;\underset{\begin{array}{c} \text{IL}-6\;\text{dissociation}\\ \text{from}\;\text{differentiated}\;\text{cells} \end{array}}{\underset{︸}{k_{r}C_{D}}}-\;\underset{\;\text{IL}-6\;\text{natural}\;\text{decay}}{\underset{︸}{\lambda_{L}L}}\;+\;\underset{\begin{array}{c} \text{IL}-6\;\text{Production}\\ \;\text{by}\;\text{tumor}\;\text{cells} \end{array}}{\underset{︸}{\rho (S+E+D)}} \end{array}$$ (7)

Eqs (8), (9) and (10) model the temporal changes in free IL-6 receptors on each of the cell types that we are considering. The first two terms in each equation are the association and dissociation of IL-6 to IL-6R. The recycling terms describe the reactions by which IL-6 is used up in the processes of mediating its cellular response, and the free receptors are recycled back to the cell surface. Following the formulation in [38], the last two terms in each equation describe the production of new free receptors as new cells are generated and the loss of these receptors as cells die. Definitions of P(⋅) and D(⋅) are provided in the following section. We note that when IL-6 binds to IL-6R, it subsequently recruits a GP130 molecule to form a ternary complex (IL-6/IL-6R/GP130) [15]. However, we do not model GP130 explicitly here, instead, we indirectly account for its role in the binding parameters and recycling parameters.

$$\begin{array}{ll} \frac{dR_{S}}{dt}= & -\underset{\begin{array}{c} \text{IL}-6\;\text{binding}\;\text{to}\\ \text{stem}\;\text{cells} \end{array}}{\underset{︸}{k_{f}LR_{S}}}\;+\;\underset{\begin{array}{c} \text{IL}-6\;\text{dissociation}\\ \text{from}\;\text{stem}\;\text{cells} \end{array}}{\underset{︸}{k_{r}C_{S}}}+\;\underset{\text{Recycling}}{\underset{︸}{k_{p}C_{S}}}\\ & +\;\;\underset{\begin{array}{c} \text{Generation}\;\text{of}\;\text{new}\;R_{S}\\ \text{via}\;\text{cell}\;\text{proliferation} \end{array}}{\underset{︸}{R_{T_{S}}P_{S}(S,\phi_{S})}}\;-\;\underset{\begin{array}{c} \text{Loss}\;\text{of}\;R_{S}\\ \text{via}\;\text{cell}\;\text{death} \end{array}}{\underset{︸}{\frac{R_{S}}{R_{S}+C_{S}}R_{T_{S}}D_{S}(S,\phi_{S})}} \end{array}$$ (8)

$$\begin{array}{ll} \frac{dR_{E}}{Et}= & -\underset{\begin{array}{c} \text{IL}-6\;\text{binding}\;\text{to}\\ \text{progenitor}\;\text{cells} \end{array}}{\underset{︸}{k_{f}LR_{E}}}\;+\;\underset{\begin{array}{c} \text{IL}-6\;\text{dissociation}\\ \text{from}\;\text{progenitor}\;\text{cells} \end{array}}{\underset{︸}{k_{r}C_{E}}}+\;\underset{\text{Recycling}}{\underset{︸}{k_{p}C_{E}}}\\ & +\;\;\underset{\begin{array}{c} \text{Generation}\;\text{of}\;\text{new}\;R_{E}\\ \text{via}\;\text{cell}\;\text{proliferation} \end{array}}{\underset{︸}{R_{T_{E}}P_{E}(E,\phi_{E})}}\;-\;\underset{\begin{array}{c} \text{Loss}\;\text{of}\;R_{E}\\ \text{via}\;\text{cell}\;\text{death} \end{array}}{\underset{︸}{\frac{R_{E}}{R_{E}+C_{E}}R_{T_{E}}D_{E}(E,\phi_{E})}} \end{array}$$ (9)

$$\begin{array}{ll} \frac{dR_{D}}{Dt}= & -\underset{\begin{array}{c} \text{IL}-6\;\text{binding}\;\text{to}\\ \text{differentiated}\;\text{cells} \end{array}}{\underset{︸}{k_{f}LR_{D}}}\;+\;\underset{\begin{array}{c} \text{IL}-6\;\text{dissociation}\\ \text{from}\;\text{differentiated}\;\text{cells} \end{array}}{\underset{︸}{k_{r}C_{D}}}+\;\underset{\text{Recycling}}{\underset{︸}{k_{p}C_{D}}}\\ & +\;\;\underset{\begin{array}{c} \text{Generation}\;\text{of}\;\text{new}\;R_{D}\\ \text{via}\;\text{cell}\;\text{proliferation} \end{array}}{\underset{︸}{R_{T_{D}}P_{D}(D,\phi_{D})}}\;-\;\underset{\begin{array}{c} \text{Loss}\;\text{of}\;R_{D}\\ \text{via}\;\text{cell}\;\text{death} \end{array}}{\underset{︸}{\frac{R_{D}}{R_{D}+C_{D}}R_{T_{D}}D_{D}(D,\phi_{D})}} \end{array}$$ (10)

Eqs (11), (12) and (13) are analogous to the ones above, as they describe changes in receptor-ligand complexes on each cell type. Similarly, in these equations, the internalization terms describe the reactions by which the complex is internalized and the free receptors are recycled to the cell surface. The last term in each equation describes the loss of these receptor complexes due to cell death.

$$\frac{dC_{S}}{dt}=\underset{\text{IL}-6\;\text{binding}\;\text{to}\;R_{S}}{\underset{︸}{k_{f}LR_{S}}}\;-\;\underset{\begin{array}{c} \text{IL}-6\;\text{dissociation}\\ \text{from}\;R_{S} \end{array}}{\underset{︸}{k_{r}C_{S}}}\;-\;\underset{\text{Internalization}}{\underset{︸}{k_{p}C_{S}}}\;-\;\underset{\begin{array}{c} \text{Loss}\;\text{of}\;C_{S}\\ \text{via}\;\text{cell}\;\text{death} \end{array}}{\underset{︸}{\frac{C_{S}}{R_{S}+C_{S}}R_{T_{S}}D_{S}(S,\phi_{S})}}$$ (11)

$$\frac{dC_{E}}{dt}=\underset{\text{IL}-6\;\text{binding}\;\text{to}\;R_{E}}{\underset{︸}{k_{f}LR_{E}}}\;-\;\underset{\begin{array}{c} \text{IL}-6\;\text{dissociation}\\ \text{from}\;R_{E} \end{array}}{\underset{︸}{k_{r}C_{E}}}\;-\;\underset{\text{Internalization}}{\underset{︸}{k_{p}C_{E}}}\;-\;\underset{\begin{array}{c} \text{Loss}\;\text{of}\;C_{E}\\ \text{via}\;\text{cell}\;\text{death} \end{array}}{\underset{︸}{\frac{C_{E}}{R_{E}+C_{E}}R_{T_{E}}D_{E}(E,\phi_{E})}}$$ (12)

$$\frac{dC_{D}}{dt}=\underset{\text{IL}-6\;\text{binding}\;\text{to}\;R_{D}}{\underset{︸}{k_{f}LR_{D}}}\;-\;\underset{\begin{array}{c} \text{IL}-6\;\text{dissociation}\\ \text{from}\;R_{D} \end{array}}{\underset{︸}{k_{r}C_{D}}}\;-\;\underset{\text{Internalization}}{\underset{︸}{k_{p}C_{D}}}\;-\;\underset{\begin{array}{c} \text{Loss}\;\text{of}\;C_{D}\\ \text{via}\;\text{cell}\;\text{death} \end{array}}{\underset{︸}{\frac{C_{D}}{R_{D}+C_{D}}R_{T_{D}}D_{D}(D,\phi_{D})}}$$ (13)

#### Proliferation and death function definitions

$P_{S}(S,\phi_{S})$, $P_{E}(E,\phi_{E})$ and $P_{D}(D,\phi_{D})$ are the rates at which new stem cells, progenitor and differentiated cells are generated, respectively. These relationships are taken directly from Eqs (1), (3) and (4) and are therefore given by

$$P_{S}(S,\phi_{S})\;=\;\alpha_{S}P_{S}\left(S,\phi_{S}\right)S$$ (14)

$$P_{E}(E,\phi_{E})\;=\;A_{in}\;\alpha_{S}\left(1-P_{S}\left(S\right)\right)S-\alpha_{E}E$$ (15)

$$P_{D}(D,\phi_{D})\;=\;A_{out}\;\alpha_{E}E$$ (16)

The second to last terms in Eqs (8), (9) and (10) assume that a total of $R_{T_{S}}$, $R_{T_{E}}$, and $R_{T_{D}}$, new free receptors are generated at the proliferation rates defined in Eqs (14)–(16); respectively.

The functions $D_{S}(S,\phi_{S})$, $D_{E}(E,\phi_{E})$ and $D_{D}(D,\phi_{D})$ are the death rates of stem cells, progenitor and differentiated cells; respectively. These relationships are taken directly from Eqs (1), (3) and (4) and are therefore given by

$$D_{S}(S,\phi_{S})\;=\;\frac{\delta_{S}}{1+\gamma_{S}\phi_{S}}S$$ (17)

$$D_{E}(E,\phi_{E})\;=\;\frac{\delta_{E}}{1+\gamma_{E}\phi_{E}}E$$ (18)

$$D_{D}(D,\phi_{D})\;=\;\frac{\delta_{D}}{1+\gamma_{D}\phi_{D}}D$$ (19)

The last terms in Eqs (8)–(10) and in Eqs (11)–(13) assume that the fraction of the total number of receptors that are either free or bound, respectively, are removed at the death rates defined in Eqs (17)–(19).

#### Notes on the model formulation

This formulation assumes that the total number (converted to fmol using molecular weight) of receptors per cell ($R_{T_{S}}$, $R_{T_{E}}$ and $R_{T_{D}}$) remains constant. This means that the total amount of IL-6R in the system should be conserved. In other words: Total IL-6R in the system that is associated with stem cells = IL-6R/cell × the number of stem cells. In terms of our variables for stem cells, this equation reduces to $R_{S}\;\left(\text{unoccupied}\;\text{IL-6R}\right)+C_{S}\;\left(\text{occupied}\;\text{IL-6R}\right)=R_{T_{S}}\times S$. We can ensure that the model equations do in fact conserve IL-6R by considering the sum of Eqs (8) and (11):

$$\frac{dR_{S}}{dt}+\frac{dC_{S}}{dt}=\;R_{T_{S}}\left(P_{S}(S,\phi_{S})-D_{S}(S,\phi_{S})\right)=\;R_{T_{S}}\frac{dS}{dt}\cdot$$

Therefore, upon integration, we have

$$\begin{array}{c} R_{S}+C_{S}=\;R_{T_{S}}S. \end{array}$$ (20)

Similarly, for progenitor and differentiated cells we have:

$$R_{E}+C_{E}=\;R_{T_{E}}E,$$ (21)

$$R_{D}+C_{D}\;=R_{T_{D}}D.$$ (22)

### Parameter values

Input parameters necessary to characterize the dynamics of the CSC, progenitor and differentiated cell pools include the cell division and death rates as well as the probability of stem and progenitor cell self-renewal. The proportion of cancer stem cells (CSCs) within a tumor varies widely among cancer types and cell lines [39]. CSCs make up only a fraction of 1% of the proliferating cells in the bone marrow and approximately 1 − 10% of the proliferating cells in epithelial cancers. Parameter values for cancer stem cells (including symmetric/asymmetric division rates) also vary widely across tumor types. In [19] the cell-cycle length is approximated around *T*_*c*_ = 25 hours which is in agreement with the result given in [40] in which *T*_*c*_ is estimated to be varying between one and two days. Therefore, for our numerical simulations we use *α*_*E*_ = ln2/1.04 and *α*_*S*_ ∈ [ln2/1.04/2, ln2/1.04]. The death rate of differentiated cancer cells, *δ*_*D*_, has varied widely in a window between 0.01 per day to 15-18 per week in previous studies [17, 27, 41, 42]. Finally, under this assumed hierarchical structure, CSCs live longer than both progenitor and differentiated caner cells [1], so the maximum death rate of progenitor and differentiated cells (*δ*_*E*_, *δ*_*D*_) is chosen to be close to but larger than the death rate of CSCs, *δ*_*S*_. The parameter values obtained from the literature are tabulated in Table 2.

**Table 2:** Parameter values taken from the literature and their sources.

Parameters	Baseline Values	Units	Reference
*A*_*out*_	2	dimensionless	[26]
*α*_*S*_	0.6	$\frac{1}{\text{day}}$	[19, 40]
*α*_*E*_	$\frac{log(2)}{1.04}$	$\frac{1}{\text{day}}$	[19, 40]
$P_{S_{min}}^{*}$	0.014	dimensionless	[17]
$P_{S_{max}}$	0.90	dimensionless	
*k*_*f*_	2.35	$\frac{1}{\text{fmol}}\frac{1}{\text{day}}$	[43]
*k*_*r*_	2.24	$\frac{1}{\text{day}}$	[15, 43]
λ	0.4152	$\frac{1}{\text{day}}$	[44]
*ρ*	7 × 10^−7^	$\frac{\text{fmol}}{\text{cell}}\frac{1}{\text{day}}$	[45–47]
$R_{T_{S}}$	1.66 × 10^−6^	$\frac{\text{fmol}}{\text{cell}}$	[48–51]
$R_{T_{E}}$	$\frac{1}{8}R_{T_{S}}$	$\frac{\text{fmol}}{\text{cell}}$	[8]
$R_{T_{D}}$	$\frac{1}{8}R_{T_{S}}$	$\frac{\text{fmol}}{\text{cell}}$	[8]

For those parameters that there was little or no published information, we compute a best fit to experimental data to obtain reasonable estimates. In the Results section, we also perform sensitivity analysis to find the most influential parameters on the tumor growth, percentage of CSCs and the fractional occupancies of bound IL-6 receptors on CSCs.

### Experimental data

**Fig 3:**
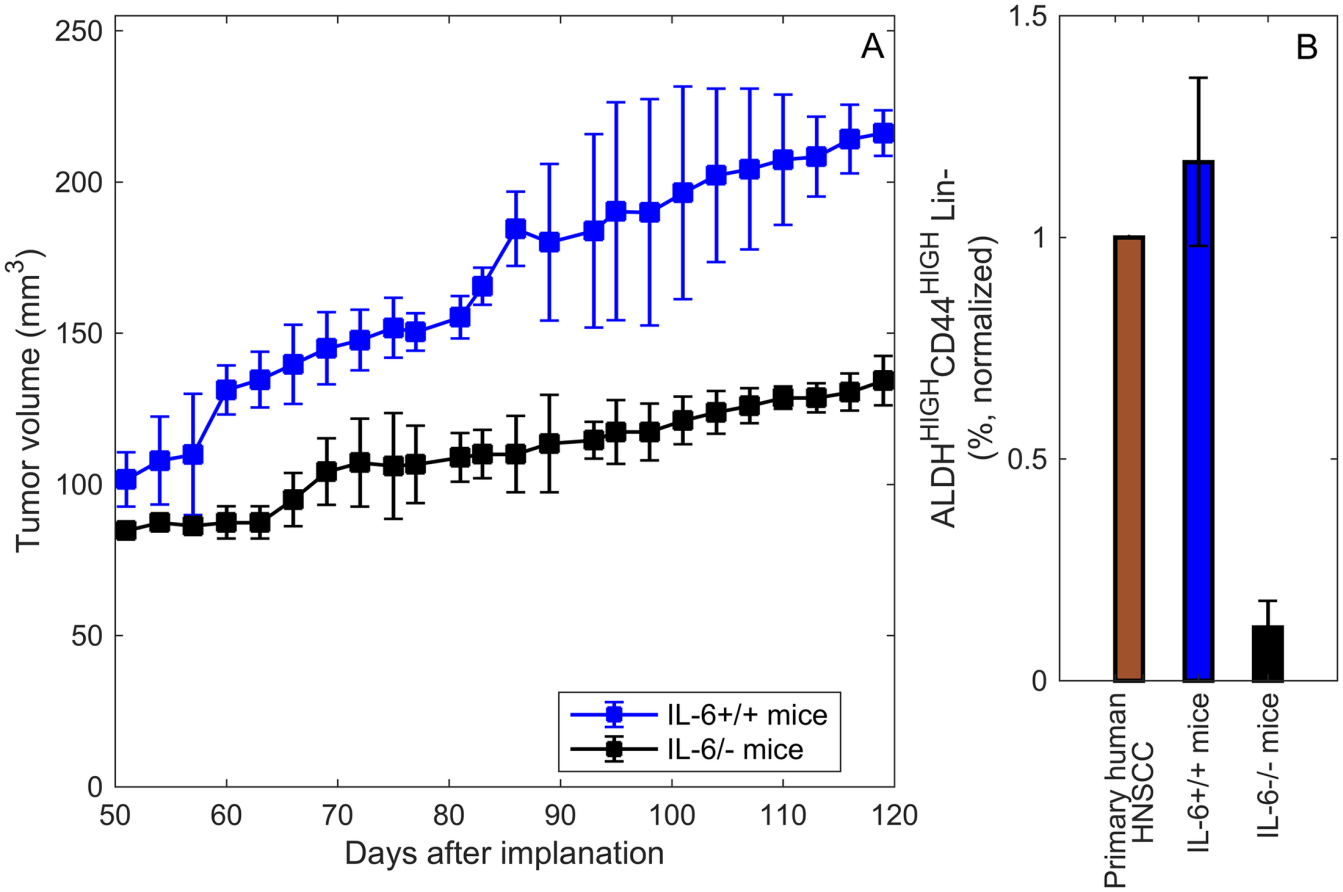
Experimental data. A: Data for tumor growth. B: Data for stem cell percentage. This data plot was redrawn from Figs 1B and 1C in [8], where they generated tumor xenografts by transplanting primary human cancer stem-like cells in SCID mice without human endothelial cells.

To begin to understand the impact of stromal IL-6 on the survival of CSCs, Krishnamurthy et al. [8] generated tumor xenografts by transplanting primary human cancer stem-like cells in severe combined immunodeficient mice. Specifically, immediately after surgical removal of the primary tumor from patients with HNSCC, ALDH^HIGH^CD44^HIGH^ cells were sorted and transplanted into IL-6 +/+ or IL-6 -/- immunodeficient mice. This approach differs from scaffold experiments where tumor xenografts, vascularized with functional human microvessels, are generated in SCID mice. In that experimental setup, human tumor cells are seeded along with human dermal microvascular endothelial cells (HDMECs) in poly(L-lactic) acid biodegradable scaffolds, resulting in the growth of human tumors with human vasculature, and an additional source of human IL-6 (the HDMECs). In the experimental setup modeled here, no human endothelial cells are implanted and the only source of human IL-6 are the tumor cells themselves. Another difference between the experimental approach modeled here and others in the literature is the use of primary tumor cells and not immortalized tumor cell lines. Fig 3 shows the relevant data presented in [8]. When 1,000 ALDH^HIGH^CD44^HIGH^ were cells transplanted into the IL-6 +/+ mice, the result was more and larger tumors as compared to the transplantation of 1000 (ALDH^HIGH^CD44^HIGH^) into IL-6 -/- deficient littermates.

#### Estimating baseline parameter values using IL6+/+ mice data

We fit the mathematical model to the IL-6+/+ mice data for primary tumor cells implanted without human endothelial cells in [8] in order to estimate the baseline parameter values for those that we could not obtain in the current literature (*A*_*in*_, $P_{N_{s}}$, *k*_*p*_, *γ*_*i*_ and *μ*). Data for tumor volume over time (day 50 through day 121) is given in Fig 3. The data-fitting process, and all the other numerical simulations of the model, uses the MATLAB ode23s solver with a time-step of one day and the initial condition given by the experiment, *S*(0) = 1000, *E*(0) = 0, *D*(0) = 0, *L*(0) = 0, *C*_*S*_(0) = 0, *C*_*E*_(0) = 0, *C*_*D*_(0) = 0. The *Monte Carlo parameter sweep method* [52] is used to minimize the Pearson *χ*^2^ statistic by comparing the extracted tumor volume from data in Fig 3 and the tumor volume predicted by the mathematical model, over 24 data time-points. **Remark:** In order to normalize the percentage of cancer stem cells by the primary human tumor stem cell percentage, it is assumed that the primary human HNSCC tumor is contained 2.1% (for a full explanation see S1 Appendix) tumor stem cells, which is normalized to 1%. The parameter values obtained via this fitting process are tabulated in Table 3.

**Table 3:** Parameter values obtained using IL-6+/+ data for primary tumor cells.

Parameters	Baseline Values	Units	Reference
*A*_*in*_	2	dimensionless	Best fit to data
*δ*_*S*_	$1.5\;\alpha_{S}P_{S_{min}}^{*}$	$\frac{1}{day}$	Assumed, based on mathematical anlysis
*δ*_*D*_	0.0612	$\frac{1}{day}$	Selected from a range of values in [17, 27, 41, 42]
*δ*_*E*_	0.0612	$\frac{1}{day}$	Assumed to be the same as *δ*_*D*_
*P*_*Ns*_	728455	dimensionless	Best fit to data
*n*	2.6	dimensionless	Assumed
*k*_*p*_	24.95	$\frac{1}{\text{day}}$	Best fit to data
*μ*	0.04	dimensionless	Best fit to data
*γ*_*i*_	2.38	dimensionless	Best fit to data

### Modeling the impact of TCZ on cancer stem cell driven tumor growth

We now extend our model by modifying it to include the therapeutic administration of Tocilizumab (TCZ), an anti-IL-6R antibody, to study the response of tumor cells to this targeted treatment.

#### Two-compartment pharmacokinetic model

Experimental evidence suggests a biphasic plasma concentration-time curve for TCZ [53]. Consequently, the following 2-compartment model is proposed to govern its pharmacokinetics. The amount of TCZ in the central compartment (systemic circulation and highly perfused tissues) is denoted by *I*_*s*_, and the amount in peripheral compartment (organs and tissues with a lower blood flow) is denoted by *I*_*p*_—see Table 4.

**Table 4:** Variables related to anti-IL-6R treatment model.

Variable	Description	Units
*I*_*s*_	Free anti-IL-6R antibody in systemic circulation	fmol
*I*_*p*_	Free anti-IL-6R antibody in peripheral compartment	fmol

The pharmacokinetic equations are given by:

$$\frac{dI_{s}}{dt}=-\underset{\text{Pharmacokinetcs}}{\underset{︸}{k_{12}\;Is+k_{21}I_{p}}}-k_{el}I_{s}+Dosing$$ (23)

$$\frac{dI_{p}}{dt}=+\;\underset{\text{Pharmacokinetcs}}{\underset{︸}{k_{12}\;Is\;-\;k_{21}I_{p}}}$$

where *k*_12_ and *k*_21_ are the transfer rate constants between the two compartments and *k*_*el*_ is the elimination rate from central compartment.

**Fig 4:**
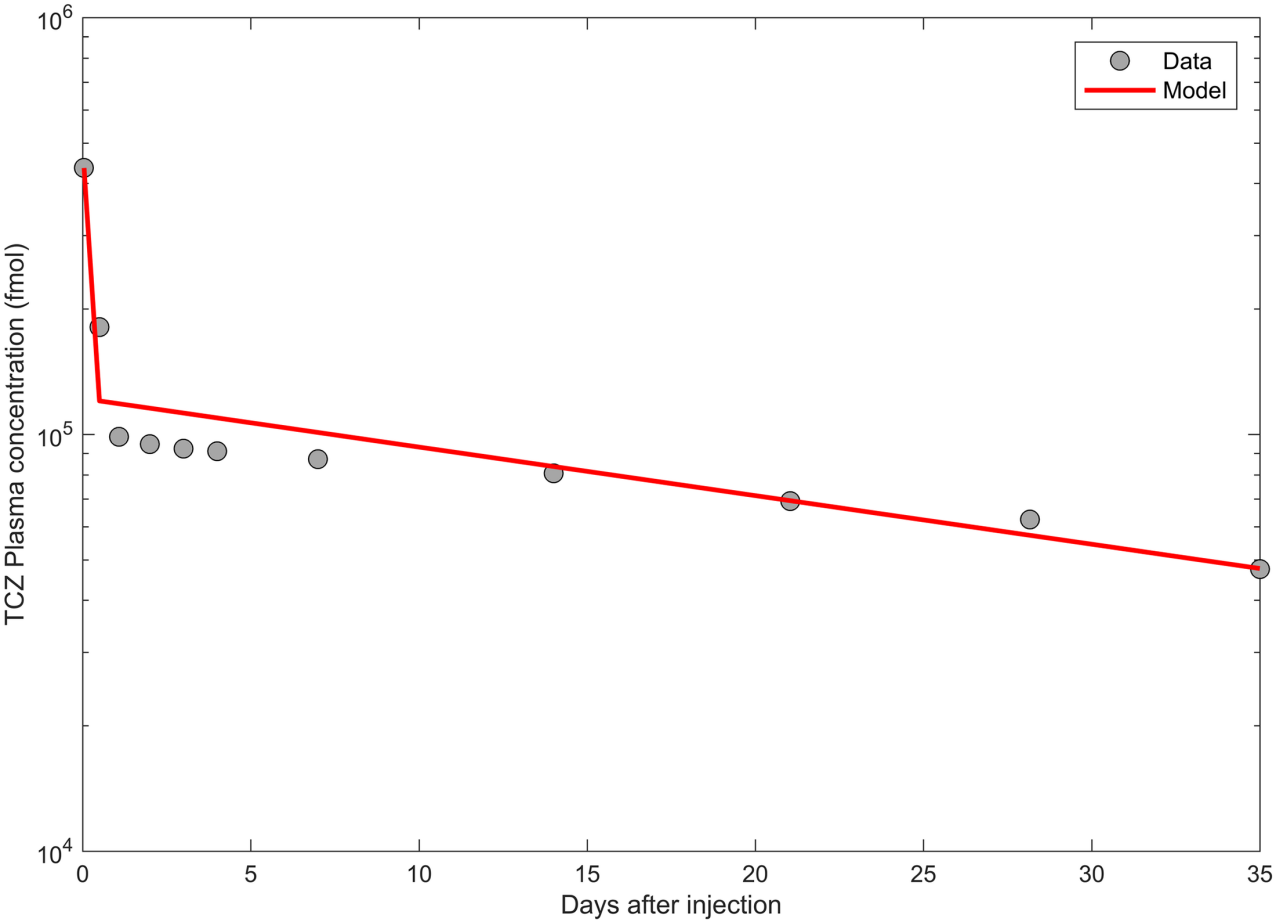
Time profiles of TCZ in plasma. The best fit of *I*_*s*_(*t*) (solid line) defined by Eq 23 is plotted together with experimental data (dots) from an in vivo study [53] of TCZ (and a PH-dependent binding variant of TCZ) in normal mice.

The pharmacokinetic constant rates (*k*_12_, *k*_21_ and *k*_*el*_) are estimated by fitting the analytical solution of *I*_*s*_(*t*) to the experimental data described in [53]. Briefly, in this experiment TCZ and a pH-dependent binding variant of TCZ, PH2, were intravenously injected at single doses of 1 mg/kg in order to calculate and compare the pharmacokinetics of TCZ and PH2 in normal mice. Plasma concentration of TCZ over time and the best fit of *I*_*s*_(*t*) to the pharmacokinetic data are shown in Fig 4 and the best-fit pharmacokinetic parameter values are tabulated in Table 5.

**Table 5:** Pharmacokinetic parameter values for the PK-model with i.v. injection.

Parameters	Values	Units	Reference
*k*_12_	14.30	day^−1^	Estimated
*k*_21_	5.55	day^−1^	Estimated
*k*_*el*_	0.004	day^−1^	Estimated

#### TCZ dosing

We define our dosing schedule based on the experiments described in [54]. Briefly, two biodegradable scaffolds seeded with human tumor and endothelial cells were transplanted in mice. When the xenograft tumors reached 200 *mm*^3^, mice were treated with 5mg/kg tocilizumab weekly. In the experiment that generated the data for our model (shown in Fig 3), scaffolds and human endothelial cells are not used. Therefore, we will administer TCZ when the xenograft tumors reach to 125 mm^3^ (the volume without scaffolds). In the experiments described in [54], TCZ is administered as a series of intraperitoneal injections. Here we assume that once injected, the drug rapidly extravasates into the systemic circulation, which approximates injection into the central compartment. Moreover, since we do not have human endothelial cells in our model, there is much less IL-6 present in the tumor environment. Therefore, we will consider TCZ administration for both a high dose of 5mg/kg and a lower dose of drug, 1mg/kg, weekly for 7 weeks.

#### Model equations related to treatment with anti-IL-6R antibody, Tocilizumab

As an anti-IL-6R antibody, TCZ binds to IL-6R on tumor cells and inhibits the formation of IL-6–IL-6R complex molecules. Soon after drug administration into the central compartment (as described in (23)), TCZ reaches the tumor environment and binds to IL-6R on tumor cells at a rate $k_{f}^{I}$, and dissociates at a rate $k_{r}^{I}$. The bound complexes of TCZ and IL-6R on stem, progenitor and differentiated cells are denoted by $C_{S}^{I}$, $C_{E}^{I}$ and $C_{D}^{I}$, respectively. Eq 24 describes the association and dissociation of TCZ in the tumor, *I*(*t*), to IL-6 cell-bound receptors on tumor cells.

$$\begin{array}{ll} \frac{dI}{dt}= & -\underset{\begin{array}{c} \text{Anti-IL6R}\;\text{binding}\;\text{to}\\ \text{stem}\;\text{cells} \end{array}}{\underset{︸}{k_{f}^{I}IR_{S}}}\;+\;\underset{\begin{array}{c} \text{Anti-IL6R}\;\text{dissociation}\\ \text{from}\;\text{stem}\;\text{cells} \end{array}}{\underset{︸}{k_{r}^{I}C_{S}^{I}}}\;-\;\underset{\begin{array}{c} \text{Anti-IL6R}\;\text{binding}\;\text{to}\\ \text{progenitor}\;\text{cells} \end{array}}{\underset{︸}{k_{f}^{I}IR_{E}}}\\ & +\underset{\begin{array}{c} \text{Anti-IL6R}\;\text{dissociation}\\ \text{from}\;\text{progenitor}\;\text{cells} \end{array}}{\underset{︸}{k_{r}^{I}C_{E}^{I}}}\;-\underset{\begin{array}{c} \text{Anti-IL6R}\;\text{binding}\;\text{to}\\ \text{differentiated}\;\text{cells} \end{array}}{\underset{︸}{k_{f}^{I}IR_{D}}}\;+\;\underset{\begin{array}{c} \text{IL}-6\;\text{dissociation}\\ \text{from}\;\text{differentiated}\;\text{cells} \end{array}}{\underset{︸}{k_{r}^{I}C_{D}^{I}}}\\ & +\underset{\;\text{Pharmacokinetics}}{\underset{︸}{k_{12}I_{s}-k_{21}I}} \end{array}$$ (24)

Underlying assumptions for this equation are: (i) the tumor resides in a pharmacokinetic compartment of its own, (ii) the binding rates are the same, independent of cell type; (iii) TCZ is transferred into the tumor (seventh term in (24)) from the systemic circulation (*I*_*s*_ obtained from (23)) at the same rate as the peripheral tissue, *k*_12_; and (iv) the tumor volume is negligible compared to the volume of mouse; therefore the amount of the drug leaking into blood stream (at the rate *k*_21_) will not affect the concentration of free TCZ in the systemic circulation. The full set of model equations, after adding treatment with TCZ, is given in the S1 Appendix.

## Results

We have developed a mathematical model for cancer stem cell-driven tumor growth that is designed to quantify the influence of tumor cell-secreted IL-6 signaling on tumor growth, cellular composition, and targeted therapy. We first compare the model predictions of (control) tumor growth in the absence of treatment to experimental data for scaffold-free primary tumor xenografts. A full description of the experimental system used to generate the data and a comparison to other experimental approaches in provided in the Methods section. We then perform a detailed sensitivity analysis of all model parameters, before accessing tumor response to targeted therapy.

### Characterizing IL-6 dependent tumor growth

**Fig 5:**
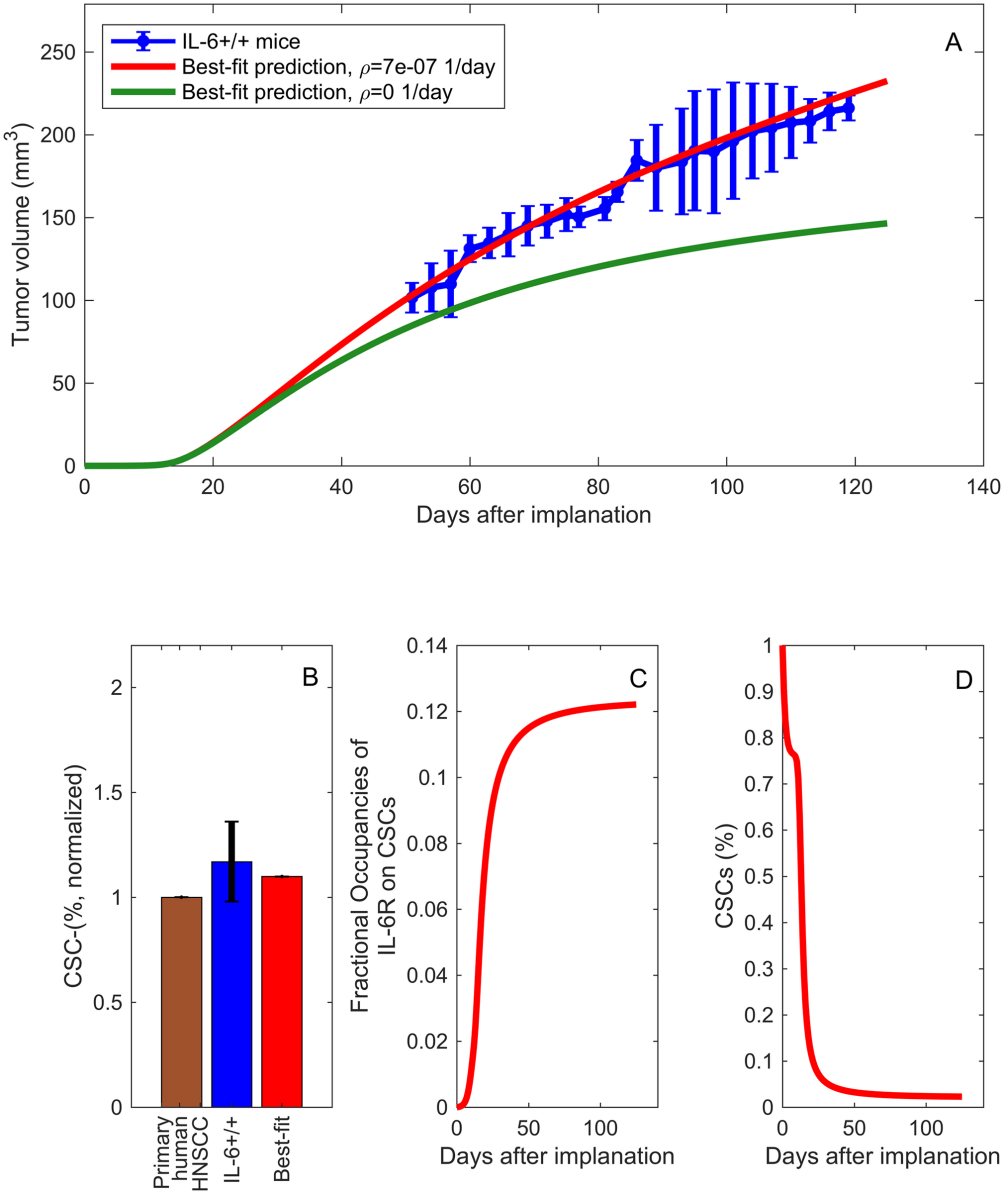
Numerical simulations of pre-treatment tumor growth, CSC percentage, and IL-6R fractional occupancy. A: Best fit of the mathematical model prediction of tumor volume over time to the IL-6+/+ data for primary tumors implanted without human endothelial cells in [8]. The green curve in (A) shows the special case in which the tumor cells are not producing IL-6, *ρ* = 0. B: Comparison of the experimentally measured percentage of CSCs in primary tumors (brown), the experimentally measured percentage of CSCs on day 121 for tumors grown in IL-6 +/+ mice (blue), and the mathematical model prediction percentage of CSCs on day 121 (red). C: Model prediction of the temporal changes in the fractional occupancy of IL-6 receptors on CSCs, *ϕ*_*S*_. D: Model prediction of the stem cell percentage over time.

Numerical simulations of pretreatment tumor growth are presented in Fig 5-A. There is a strong correlation between the model output (red) and the experimental data in [8] (blue). The green line in Fig 5-A represents the tumor volume over time when tumor cells do not produce IL-6, thereby showing how much even low secretion rates of IL-6 ($\rho =7\times 10^{-7}\frac{\text{fMol}}{\text{cell}\times \text{day}}$) influence tumor growth. In addition, we use the best-fit parameter values to predict the percentage of CSCs on the last day of the experiment (see the Methods section for details on the experimental design). Fig 5-B shows the experimentally measured percentage of CSCs in primary tumors (brown), the experimentally measured percentage of CSCs on day 121 for tumors grown in IL-6 +/+ mice (blue), along with our mathematical model prediction (red). The model is able to accurately capture the correct proportion of stem cells and Fig 5-D shows how the stem cell percentage evolves over time.

The first step in the IL-6 signal transduction pathway is to binding to IL-6R. The IL-6-IL-6R complex then recruits GP130. The complex of IL-6-IL-6R-GP130 activates signaling pathways (such as STAT3) [55, 56] that play a critical role in the self-renewal and survival of CSCs. Therefore, the fractional occupancies of bound receptors can be a useful tool for quantifying the influence of tumor cell-secreted IL-6 on the tumorigenic potential of CSCs and subsequently on tumor growth dynamics. Fig 5-C plots the fractional occupancy of IL-6R on CSCs over time for our baseline level of tumor secretion of IL-6. The model suggests that a fractional occupancy of 12% on CSCs is sufficient to result in the experimentally observed tumor growth rate. In fact, because endothelial cells can secrete higher levels of IL-6 than tumor cells [8], if we were to add endothelial cells to our model then we would expect even greater interdependencies among IL-6, tumor growth dynamics and the tumorigenic potential of CSCs.

**Fig 6:**
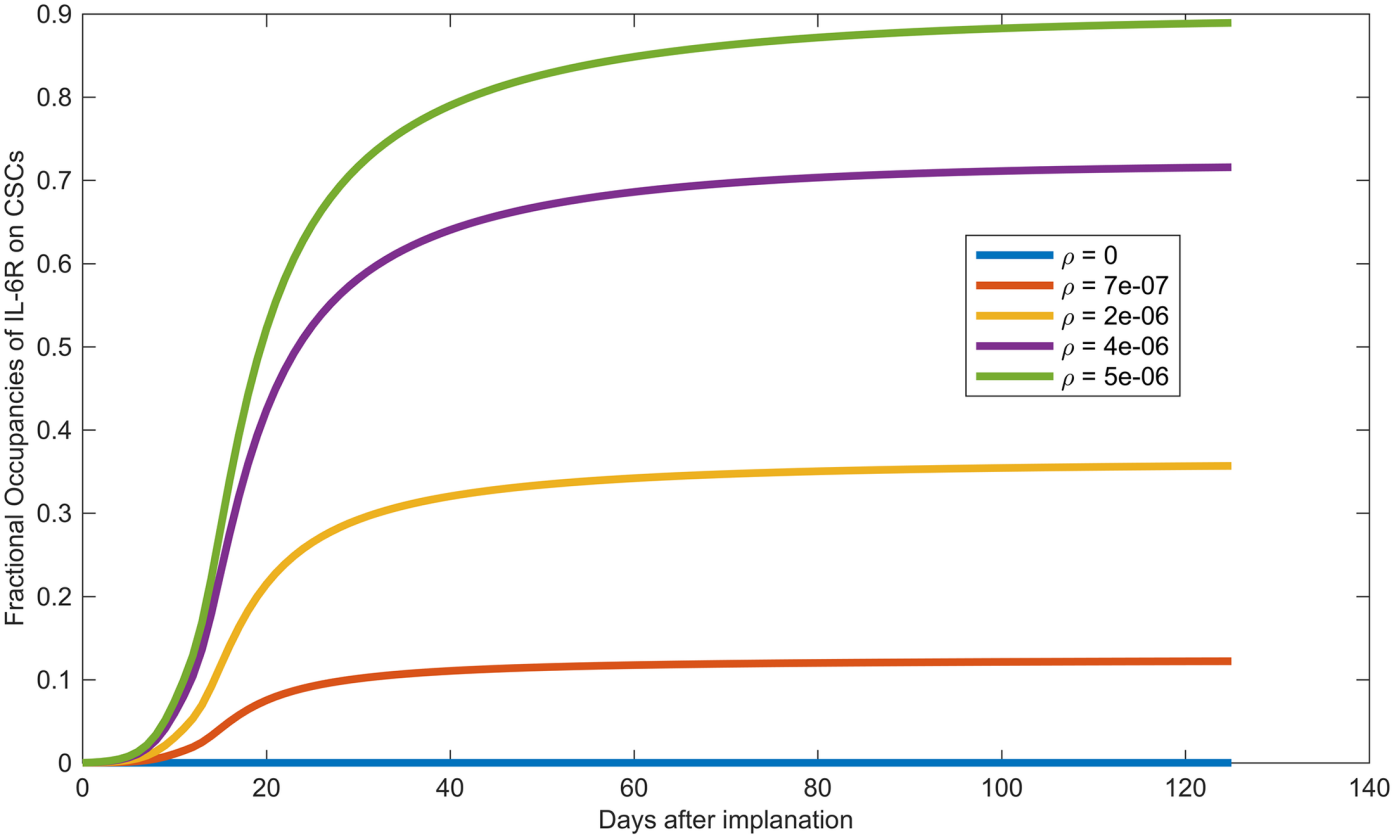
IL-6R fractional occupancy. Model prediction of the temporal changes in the factional occupancy of IL-6 receptors on CSCs, *ϕ*_*S*_, as the IL-6 secretion rate by tumor cells, *ρ*, varies from its baseline value (*ρ* = 7 × 10^−7^ fmol/cell/day) to the value that leads to 90% fractional occupancy (5.35 × 10^−6^ fmol/cell/day).

Values for the IL-6 secretion rate by tumor cells, *ρ*, vary quite widely in the literature [45–47]. Our baseline value of *ρ* = 7×10^−7^ corresponds to 200 pg per ml per 10^6^ HNSCC cells per day, which is within the ranges reported in [45–47]. Fig 6 shows that if we keep all the other parameters at their baseline values provided in Tables 2 and 3, while varying the secretion rate of IL-6, *ρ*, then relatively small increases in *ρ* (from *ρ* = 7×10^−7^ to 5.35×10^−6^ fmol/cell/day) lead to 90% fractional occupancy. This supports the idea that an IL-6 antagonist could temper the effects of IL-6-induced pathways, thereby impeding tumor growth.

#### Sensitivity analysis

We use uncertainty quantification and sensitivity analysis to determine which parameters (see Table 1 for definitions of the model variables and Tables 2 and 3 for a list of baseline parameters) are the most influential on the tumor volume, $TV=\frac{S(t)+E(t)+D(t)}{10^{6}}$, percentage of cancer stem cells, $\Omega =\frac{S(t)}{S(t)+E(t)+D(t)}$ and the fraction of occupied receptors on CSCs, *ϕ*_*S*_ given by Eq 2, at days 15, 50, 90 and 120. The method we employ is a global sensitivity analysis that uses Latin Hypercube Sampling (LHS) along with Partial Rank Correlation Coefficient (PRCC) to assess the sensitivity of the output of interest (tumor volume, percentage of cancer stem cells, and the fraction of occupied bound receptors) to each of the parameters at the given time-points [57–59]. We use the best-fit parameter values as the baseline to calculate LHS PRCC values and ±10% of the best-fit parameter values as the range.

**Fig 7:**
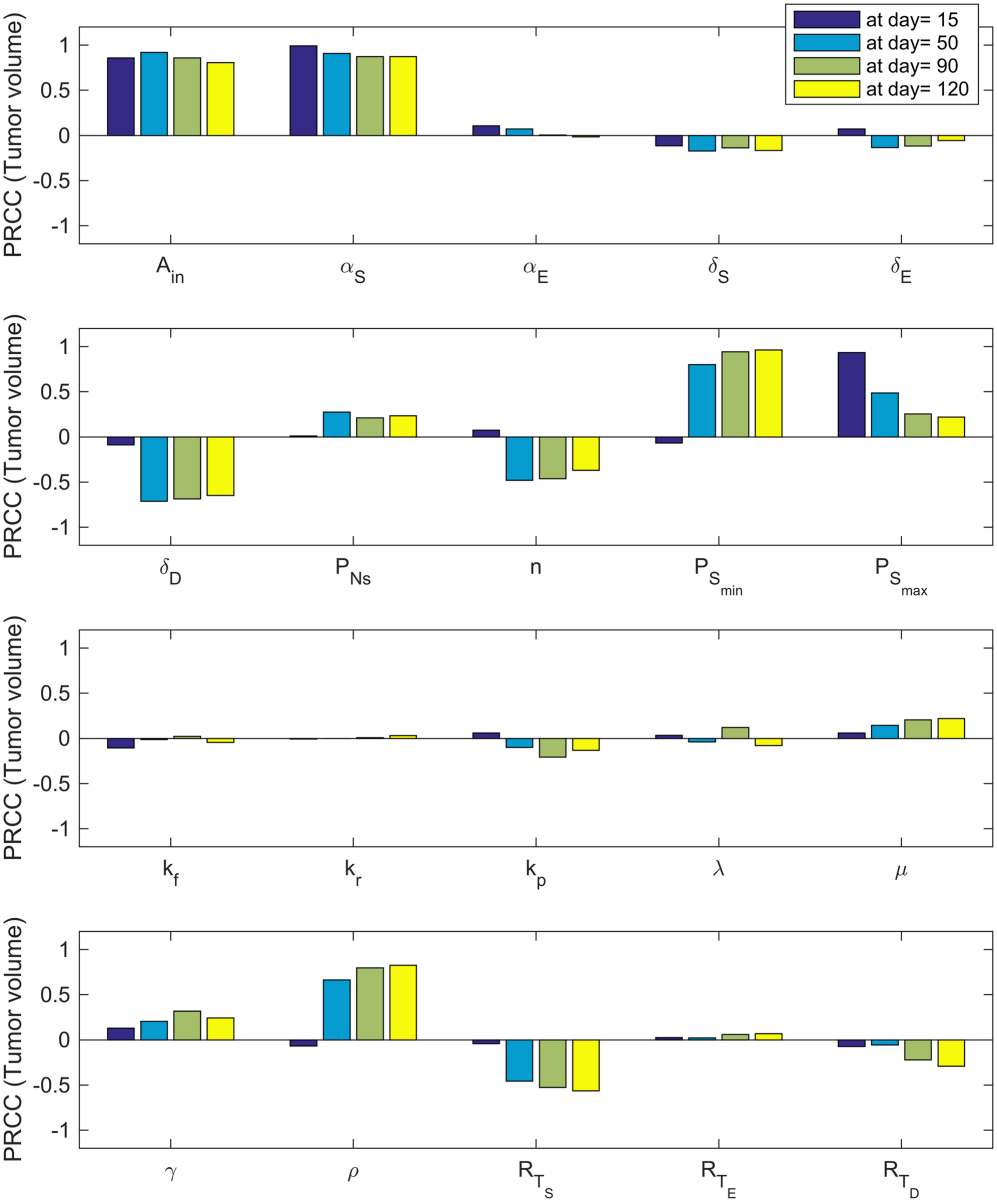
Tumor volume sensitivity. PRCC values for the parameters using the tumor volume as the output of interest.

**Fig 8:**
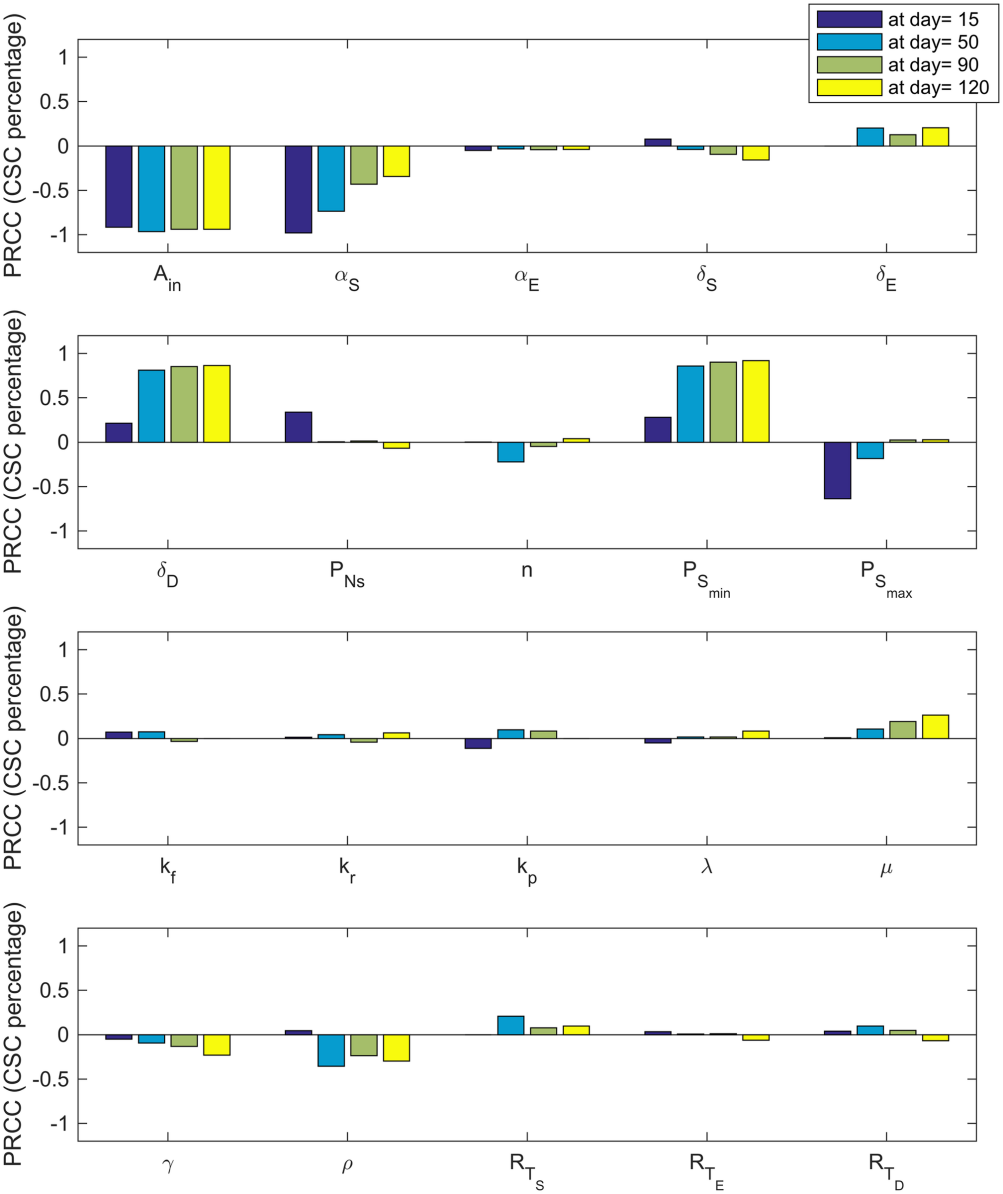
CSC percentage sensitivity. PRCC values for the parameters using the percentage of cancer stem cells as the output of interest.

**Fig 9:**
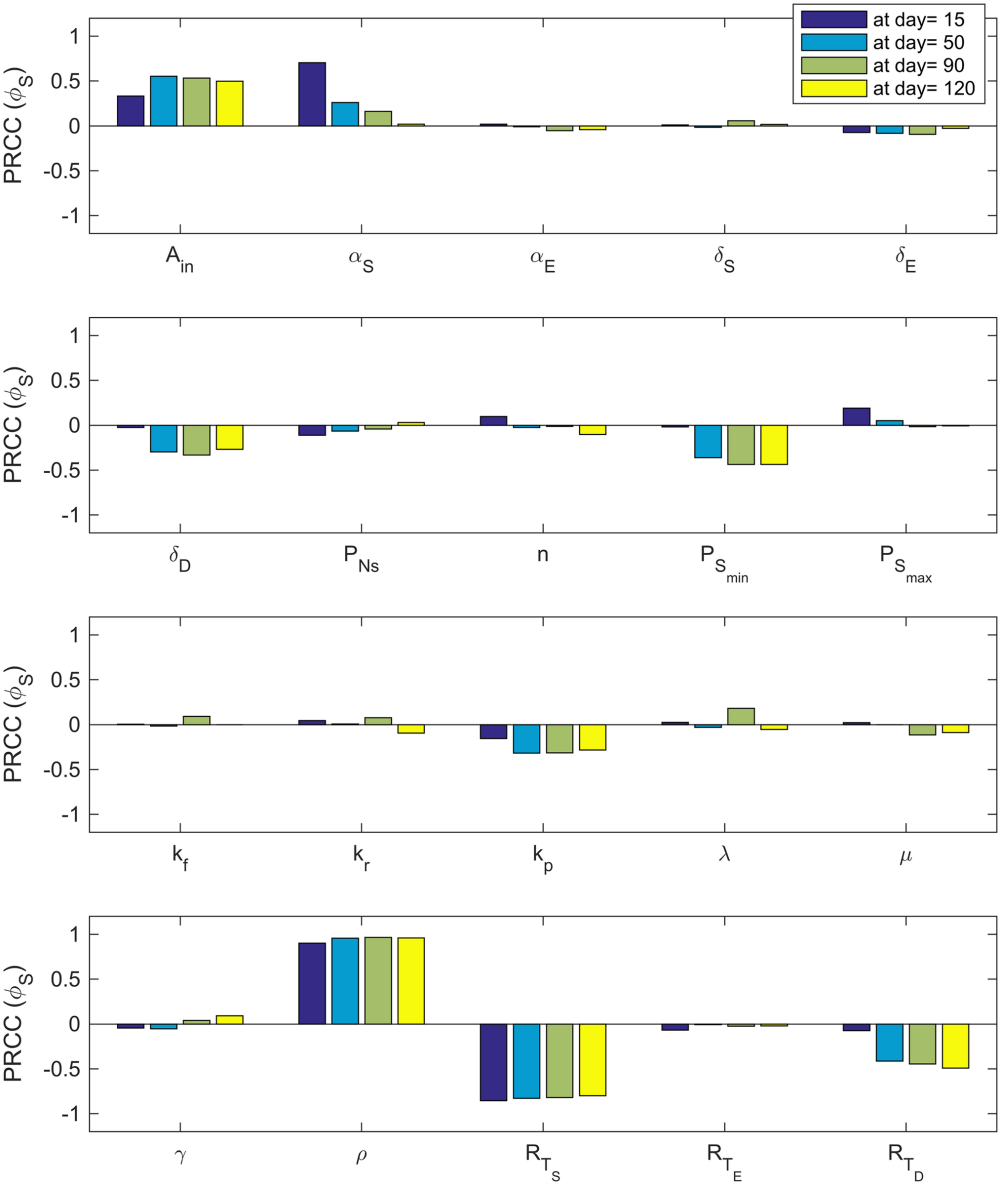
IL-6R sensitivity/PRCC values for the parameters using the fractional occupancy of IL-6R on CSCs as the output of interest.

As depicted in Figs 7–9, sensitivity analysis reveals that in some cases, parameter sensitivity varies as the tumor grows. For instance, since |*PRCC*(*TV*, *A*_*in*_)| ≈ 1 and |*PRCC*(*TV*, *α*_*S*_)| ≈ 1 (Fig 7), tumor volume is highly sensitive to the relatively small changes in both the amplification factor, *A*_*in*_, and the stem cell proliferation rate, *α*_*S*_, at all the times. However, the tumor volume becomes less and less sensitive to the parameter $P_{S_{max}}$ as tumor volume increases. Other parameters with a large influence on the tumor volume are the death rate of the terminally differentiated cancer cells, *δ*_*D*_; the minimum probability of CSC self-renewal, $P_{S_{min}}^{*}$; the production rate of IL-6 by tumor cells, *ρ*; and the total number of IL-6 receptors on CSCS, $R_{T_{S}}$. These results highlight how critically important CSC dynamics are for driving tumor growth. Interestingly, the only influential parameter not related to CSCs and IL-6 is the maximum death rate of the terminally differentiated cancer cells.

PRCC values given in Fig 8 show that percentage of CSCs within the tumor is also highly influenced by the amplification factor, *A*_*in*_, at all times. The death rate of the terminally differentiated cancer cells, *δ*_*D*_ and the minimum probability of CSC self-renewal, $P_{S_{min}}^{*}$, are most influential at later times, while the stem cell proliferation rate, *α*_*S*_ becomes less influential as the tumor grows larger.

Finally, PRCC values for parameters using the fractional occupancy, *ϕ*_*S*_, as the output of interest (Fig 9) reveals that *A*_*in*_, *ρ*, and $R_{T_{S}}$ are consistently the most influential parameters. Again, *α*_*S*_ is influential early in tumor growth, but loses its impact for later times.

### Predicting the effect of TCZ therapy

**Fig 10:**
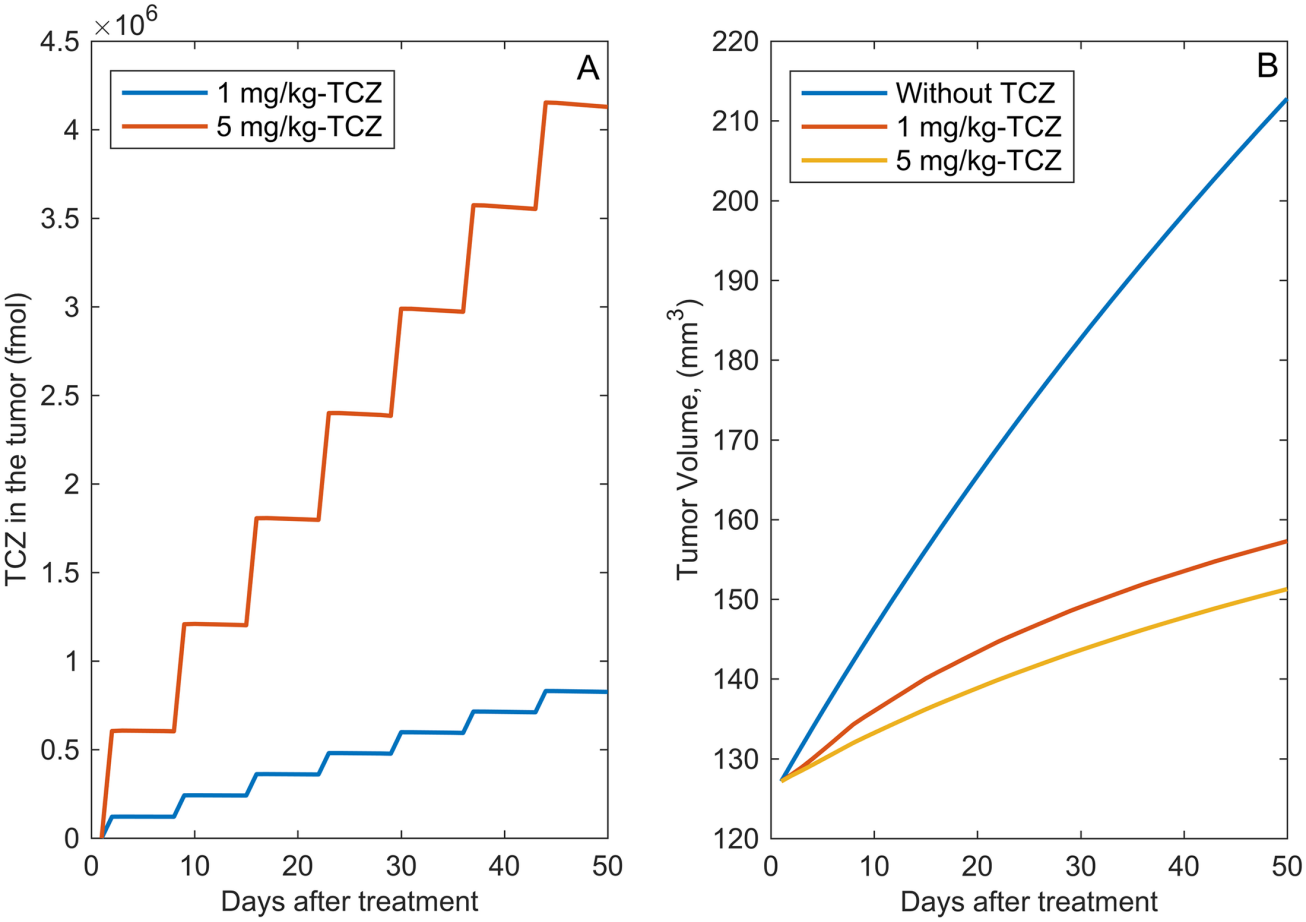
Numerical simulations of TCZ treatment. A: The amount of TCZ within the tumor during 7 weeks of treatment. B: Model predictions of tumor volume vs. time after treatment with TCZ. 1mg/kg or 5 mg/kg of TCZ is administered weekly when tumor reaches 125 mm^3^.

We used the treatment model (see S1 Appendix) together with the parameter values listed in Tables 2, 3 and 5 to predict tumor response to weekly administration of 1mg/kg or 5mg/kg of TCZ for 7 weeks. Fig 10-A shows the model predictions for the amount of TCZ within the tumor for the two different doses. Our model predicts a 25% and 28% reduction in the tumor volume as compared with tumor volume without treatment for the two different dosing strategies (Fig 10-B). This result is very similar to the experimentally observed tumor reduction shown in [54] for UM-HMC-3B, salivary gland cancer xenografts.

#### Small doses of TCZ are sufficient to outcompete IL-6

**Fig 11:**
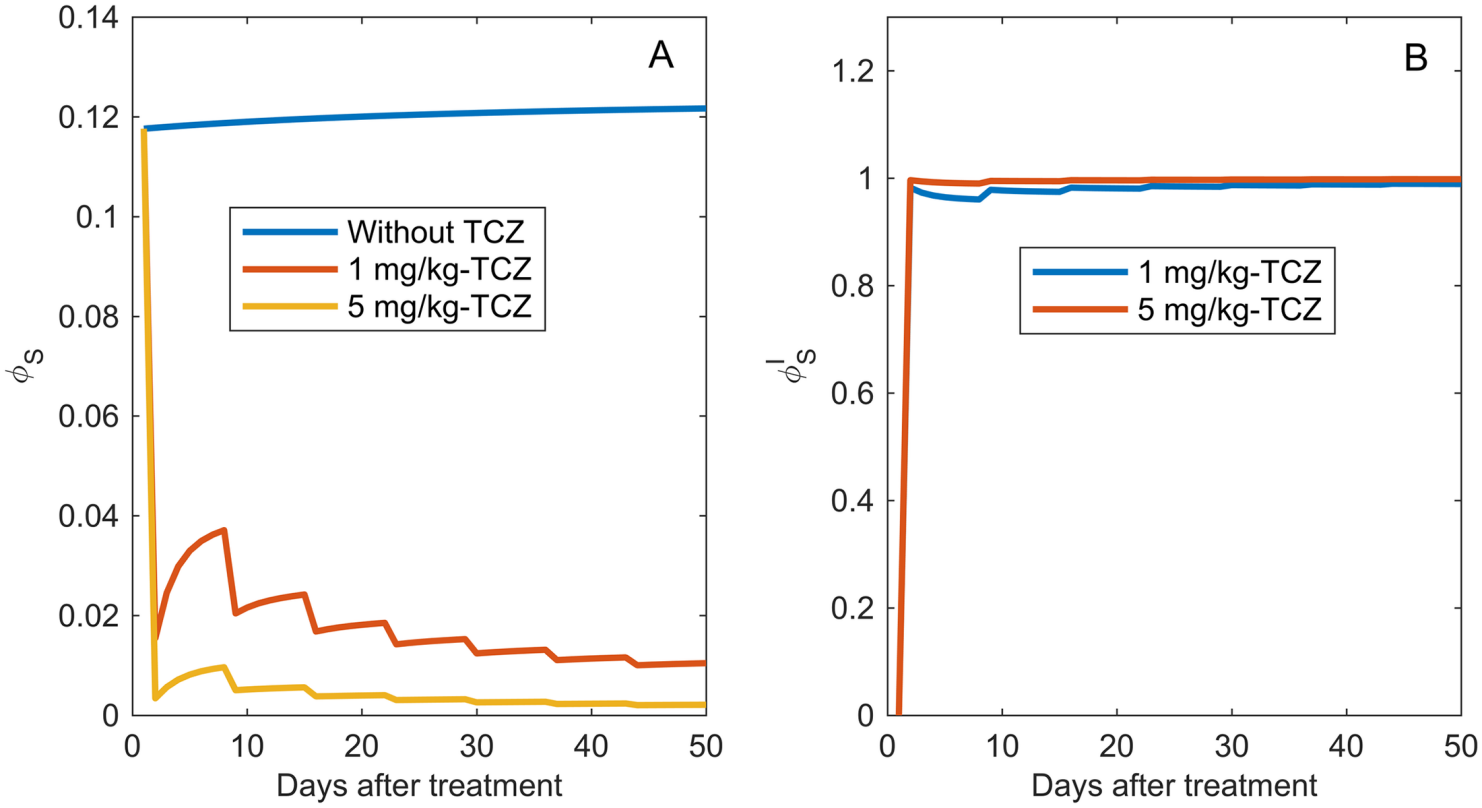
IL-6R occupancy post-treatment. A: Model prediction of the fraction of IL-6 receptors on CSCs over time that are occupied by IL-6, *ϕ*_*S*_, for the control case (no treatment, blue), 1 mg/kg TCZ (red), and 5 mg/kg TCZ (yellow). B: Model prediction of the fraction of IL-6 receptors on CSCs over time that are occupied by TCZ, *ϕ*_*I*_, for doses of 1 mg/kg TCZ (blue), and 5 mg/kg TCZ (red).

The reduction in tumor growth described above is due to the fact that TCZ competes with IL-6 for IL-6R, which results in a sudden decrease in the number of the IL-6–IL-6R signaling complexes. As shown in Fig 11, after treatment there is an 80-90% decrease in the fraction of IL-6R occupied by IL-6 on tumor cells. Fig 11 also shows that administration of doses as small as 1mg/kg of TCZ is sufficient for saturating IL-6R with TCZ molecules under these experimental conditions.

#### The effect TCZ on tumor cell death is more pronounced than its effect on CSC self-renewal

**Fig 12:**
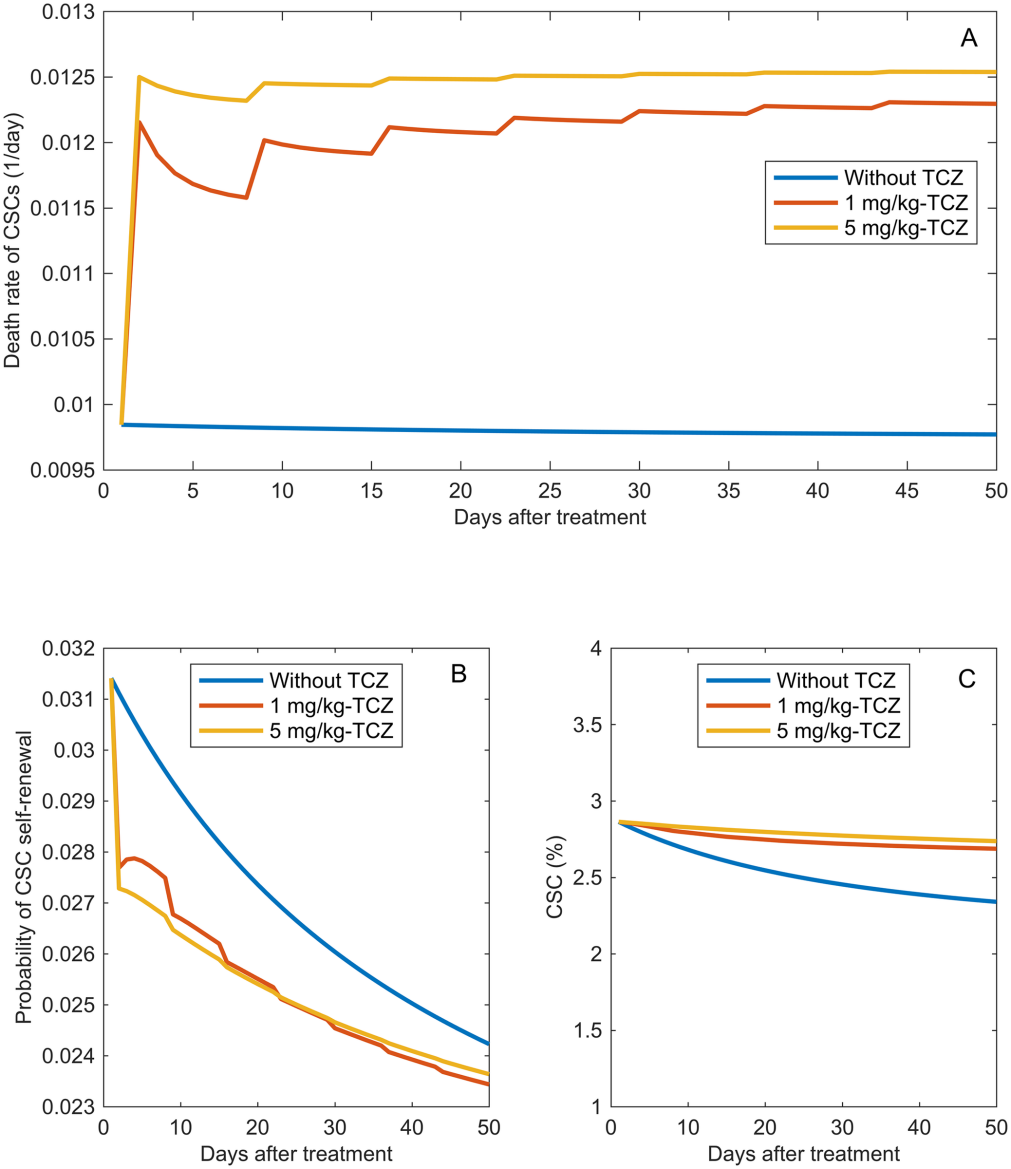
TCZ impact on CSC death, self-renewal, and percentage. Model predictions of the temporal impact of administering 1mg/kg or 5mg/kg of TCZ on the A: death rate of CSCs, B: probability of CSC self-renewal and C: percentage of CSCs within the tumor. As mentioned in the Section 2.2, these predictions are based on training the model with data from [8] were primary tumor cells were transplanted into IL6 +/+ mice without the addition of human endothelial cells.

Administration of TCZ interferes with the IL-6-mediated pathways that enhance the survival properties of all cancer cells and the self-renewal properties of CSCs. Specifically, the TCZ mediated reduction in IL-6-IL-6R signaling complexes decreases the pro-survival effects of IL-6, which results in an increase in the death rates of tumor cells. Our model predicts an increase of approximately 24-27% in the death rates of CSCs (Fig 12-A). Progenitor cells and terminally differentiated cells follow the same trend. The same mechanism also results in a decrease in the probability of CSC self-renewal. Fig 12-B shows that TCZ causes an early reduction of 12-13% in the probability of CSC self-renewal. However, in the later weeks of treatment this difference decreases to 2-4%. This marginal impact of TCZ on the self-renewal probability is likely due to the tendency of CSCs to quickly reach to their equilibrium level (see Fig 5-D).

Changes in self-renewal and death rates of CSCs alter the fraction of CSCs post-treatment. Our model predicts that there is a small increase in the percentage of CSCs after treatment with TCZ (Fig 12-C). That is, the effect of TCZ on tumor volume is characterized by overall tumor reduction, but a final tumor composition that has a slightly larger proportion of CSCs. It is important to note that the *number* of CSCs within the tumor drops significantly post treatment; under this particular set of experimental conditions, the model predicts that impact of TCZ on the number of CSCs is more likely caused by its effect on cell survival as opposed to self-renewal. IL-6 also impacts the proliferation and survival of bulk cells, so their number also decreases also when TCZ is administered. The model predicts an approximately 20% percent decrease in the number of CSCs post therapy and a 25% decrease in tumor volume. We define CSC proportion as the total number of CSC divided by the total number of cancer cells. Both the numerator and denominator in this equation are decreasing due to therapy, so the overall therapeutic approach is successful.

**Fig 13:**
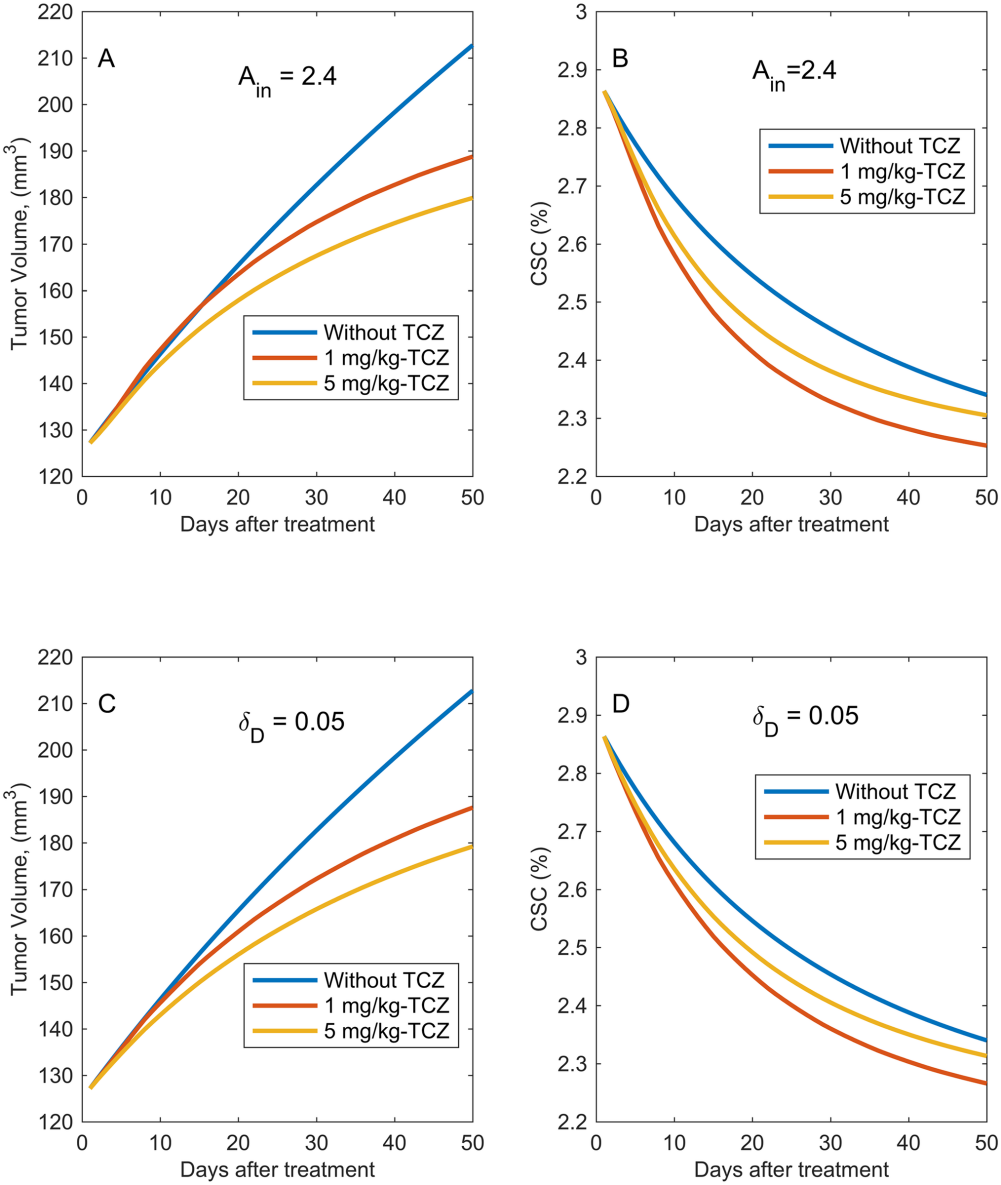
Impact of the amplification factor, *A*_*in*_ and the differentiated cell death rate, *δ*_*D*_ on tumor growth. Model predictions of the tumor volume vs. time for the control cases as well as for treatment with 1 or 5 mg/kg TCZ when A: the amplification factor, *A*_*in*_, is slightly increased from its baseline value and when C: the differentiated cell death rate, *δ*_*D*_ is slightly decreased from baseline. Model predictions of the CSC percentage vs time for the control cases as well as for treatment with 1 or 5 mg/kg TCZ when B: the amplification factor, *A*_*in*_, is slightly increased and when D: the differentiated cell death rate, *δ*_*D*_ is slightly decreased (D).

Interestingly, the minor increase in the percentage of CSCs predicted by our model is somewhat comparable to the results reported in [54] where they showed that TCZ has mixed effects on the fraction of CSCs post therapy. Given these varied results for the influence of TCZ of CSC percentage, we repeated the sensitivity analysis described above and considered PRCC values both before and after the first dose of TCZ. Comparing the most influential parameters before and after treatment, suggests that administration of the drug does not change the set of parameters to which the percentage of CSCs is most sensitive. The most influential parameters remain *A*_*in*_, *δ*_*D*_ and $P_{S_{min}}^{*}$. Fig 13 shows that even slightly increasing the amplification factor, *A*_*in*_, by as little as 20% after treatment begins also reduces the percentage of CSCs post therapy. Decreasing the maximum death rate for the terminally differentiated cancer cells by as little as 20% has the same effect on the final percentage of CSCs.

Given that many regulatory mechanisms including localized signaling cells and the extracellular space within the stem cell niche controls stem-cell fate, the simulations above suggest that the mixed results observed for the CSC % might be due to the fact that TCZ alters the balance of stem cell niche signaling in a way that impacts critical parameters such as the amplification factor and the death rate of differentiated cells. There is evidence that the number of TA divisions (*A*_*in*_) is flexible in various tissues and can respond to extracellular signals [60, 61]. It is also possible, with the experimental design that we are modeling, that after drug administration, more free human IL-6 in the tumor environment will be available for binding to murine IL-6R, leading to angiogenesis and other events that can provide better conditions for cell survival thereby impacting *δ*_*D*_.

#### The frequency of dosing does not significantly impact tumor response to TCZ

**Fig 14:**
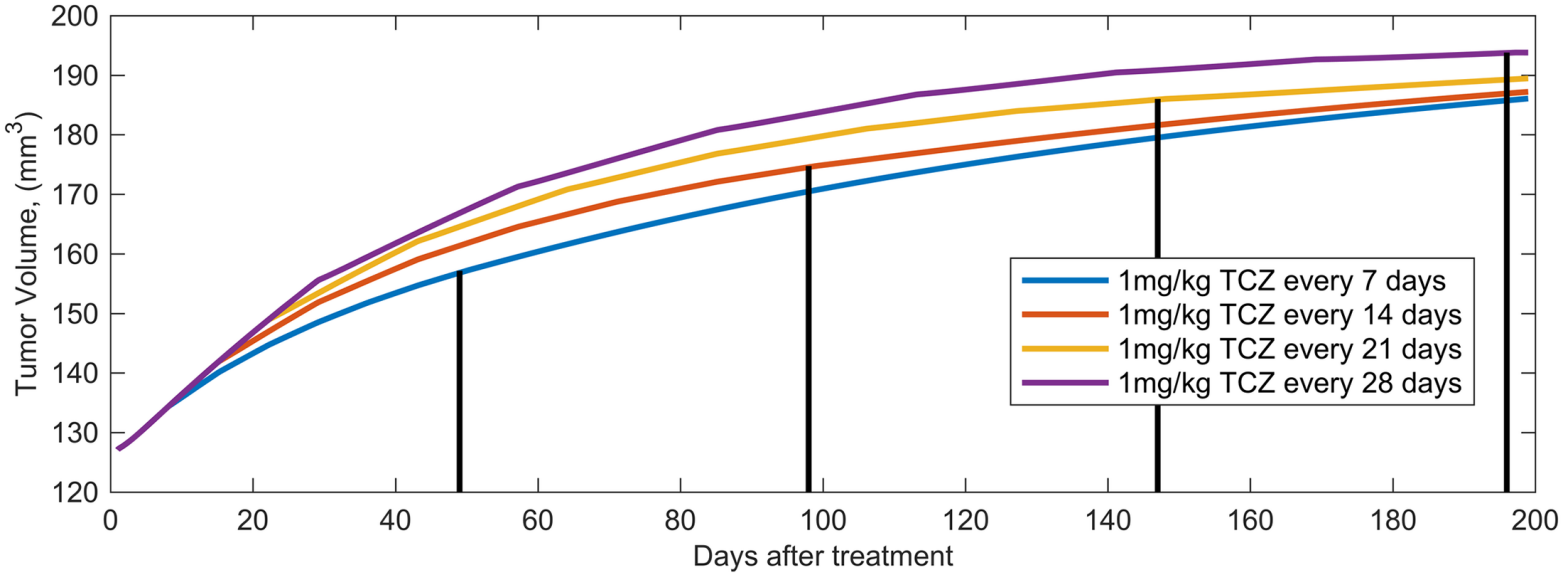
Impact of dose frequency. Model predictions of the tumor volume vs. time for the control case (no treatment) as well as for treatment with 1 mg/kg TCZ administered every 7, 14, 21 and 28 days. The line shows the first day of the final dose for each treatment schedule.

In the simulations above, TCZ was administered in doses of 1 or 5 mg/kg weekly for 7 weeks. Eventually, we will use modified and extended versions of this model to optimize the timing of combination therapies that deliver TCZ along with cytotoxic chemotherapeutic agents like cisplatin. However, even for delivery as a single agent, the effect dose frequency has on tumor response is an open question. Furthermore, anti-cancer therapies may be selected based on the convenience of administration to patients, so understanding the differential effect of various dosing schedules is imperative. Therefor, in Fig 14, we used the model to predict the effect of administering TCZ every 14, 21 and 28 days. It is important to note that the rate of decline in plasma concentrations due to the process of drug redistribution from the central to the peripheral compartment is relatively fast for TCZ as is evidenced by its *α*-half life of less than 24 hours. However, the rate of decline due to the process of drug elimination is very slow for TCZ, which can be seen by how long it lasts in the systemic circulation (Fig 14) and by considering the *β*-half life, which is approximately six months. Therefore, there are still substantial amounts of TCZ available within our longer dosing windows.

Fig 14 shows that increasing the time between doses does not lead to large increases in tumor volume. In fact, there is little difference in tumor response among all of the dosing schedules tested. Together these results suggest that administering TCZ every 3 or 4 weeks might be preferable to weekly administration as this is clinically more desirable and it leads to only minor changes in tumor volume.

## Discussion

Tumors are a heterogeneous mixture of cells with different morphological, phenotypic and molecular properties [62]. One type of tumor heterogeneity stems from the fact that that a limited proportion of cancer cells (cancer stem cells) are capable of tumor initiation [1–5]. In order to improve patient outcomes for stem-cell driven cancers, substantial research is being focused on understanding the molecular biology of cancer stem cell (CSC) self-renewal in an attempt to selectively target pathways that give them their tumorigenic potential. Studies of HNSCC have shown that these cancers express high levels of Interleukin-6 (IL-6), and both autocrine and paracrine signaling have been hypothesized as mechanisms for their IL-6 mediated growth dynamics [8, 63, 64]. IL-6 is also a key contributor in the production of CSCs and in their dynamic equilibrium with non-stem cells [2]. Taken together, this data has prompted investigations of the therapeutic inhibition of the IL-6 pathway by humanized anti-IL-6 antibodies [65] and IL-6 receptor antagonists [54, 66]. Recent studies show that IL-6 ligand and receptor targeted therapies can inhibit the survival of cancer stem cells, delay tumor initiation, prevent tumor recurrence, and enhance the anti-tumor effect of conventional chemotherapy [8, 54, 65].

In this paper we developed an experimentally based mathematical model for the growth dynamics of HNSCC tumor xenografts, which were generated by transplanting a small number of primary human cancer stem cells (ALDH^HIGH^CD44^HIGH^) in IL-6+/+ immunodeficient mice. The model includes the effects of human tumor cell-secreted IL-6 signaling on tumor cell survival and proliferation, and also captures the effect of IL-6 on the probability of self-renewal for cancer stem cells. While numerous models of cancer stem cell driven tumor growth and related treatments have been proposed (see [17] for a review), few work across the scales proposed here, from signal initiation to tissue level cancer growth. This model is the first of its kind in that it incorporates the molecular details of IL-6 binding to its natural receptor, IL-6R and allows for the quantification of the temporal changes fractional occupancies of bound receptors and their impact on tumor growth dynamics. The model predictions suggest that a fractional occupancy of 12% on CSCs is sufficient to result in the experimentally observed tumor growth rate for these primary tumor xenografts. In the experimental system that is modeled here, the only source of human IL-6 is the cancer cells themselves, as murine IL-6 does not bind to human IL-6R [67] and cannot directly initiate signals on human cells. We tested the situation where tumors cells were not able to produce IL-6 and found the final tumor sizes to be approximately 45% smaller without direct IL-6 signaling.

There is evidence that human endothelial cells, key players in tumor angiogenesis, can secrete even higher levels of IL-6 than tumor cells [26]. This implies that the 12% fractional occupancies predicted by our model leaves room for increases in proportion of bound IL-6R and more aggressive tumor growth when endothelial cells add to the amount of IL-6 available in the tumor microenvironment. There are several experimental models where human dermal microvascular endothelial cells (HDMECs) are transplanted in mice along with human tumor cells using biodegradable, polymer scaffolds [8, 54, 63, 68]. We have developed a preliminary mathematical model of this experimental paradigm and our early results show high fractional occupancies of IL-6R lead to even greater interdependencies among IL-6, tumor growth dynamics and the tumorigenic potential of CSCs.

According to the cancer stem cell hypothesis, only a small minority of cells within the tumor should be tumor-initiating cells. Our model is also able to accurately capture the correct, experimentally observed tumor composition consisting of a very small proportion of cancer stem cells. A simple and general model of the cancer stem cell hypothesis was developed to track cell state transitions. Following [26, 27], each non-cancer stem cell is amplified by a factor, *A*_*in*_ upon entry into the progenitor cell pool. Interestingly, the best fit to the experimental data was obtained with a value of *A*_*in*_ = 2, which implies that each progenitor or transient amplifying cell (TAC) only undergoes a one round of amplification before differentiating into a terminally differentiated cell (see derivation in the Materials and methods section). The fact that our model predicts that little amplification occurs is not surprising because we are using data from human primary tumor cell xenografts, as opposed to cancer cell lines. Also, in the experimental system modeled here that does not include microenvironmental production of IL-6 by endothelial cells, tumors grow relatively slowly, which is consistent with limited amplification. Cancer stem cell proportion can vary related to the local environment. For example (see [8]) the inclusion of IL-6 producing endothelial cells increases the growth rate and the proportion of cancer stem cells. Our preliminary simulations of the experimental system that includes human endothelial cells show that increased amplification, along with additional IL-6R engagement, can predict the faster tumor growth dynamics that are observed with that experimental approach.

It also important to note that although we model the traditional cancer stem cell ideology (CSC → Progenitor cell → Terminal cell), which has been published for several solid tumor types [26, 69–71], transient amplifying cells are somewhat challenging to fit into the rubric of HNSCC cancer stem cells due to limitations in the identification of markers for these cells. Here we consider cancer stem cells to be ALDH^HIGH^CD44^HIGH^. Therefore, our three cellular compartment approach could be envisioned to consist of: CD44^HIGH^ cells as the population of stem cells, various combinations of ALDH^HIGH^CD44^LOW^ and ALDH^LOW^CD44^HIGH^ cells as the pool of mixed cells, and finally ALDH^LOW^CD44^LOW^ cells. Our model predicts an amplification factor of 2 for the mixed cell pool. In terms of the previously mentioned markers for HNSCC, our modeling paradigm can be thought of as each ALDH^HIGH^CD44^HIGH^ cell (S) asymmetrically dividing (with some probability) to become one ALDH^HIGH^CD44^LOW^ or one ALDH^LOW^CD44^HIGH^ (P) cell. That newly generated cell then divides to give rise to 2 new mixed cells. Those two mixed cells then eventually divide to produce four terminally differentiated, ALDH^LOW^CD44^LOW^ (D), cells. This limited amount of amplification predicted for primary tumor cells leads to relatively slow tumor growth and a tumor composition that consists of mostly differentiated cells. Interestingly, preliminary simulations of the experimental system that includes SCC cell lines and human endothelial cells show that increased amplification and IL-6R engagement can lead not only lead to faster tumor growth, but can also change the tumor composition to be dominated by progenitor cells. It is important to note that this is simply one way to interpret the modeling results. Transitions between stem and non-stem states have not been experimentally established temporally or in response therapy. These are topics that we will tackle in future modeling and experimental studies.

We also used the model to predict tumor response to administration of the humanized IL-6R monoclonal antibody, tocilizumab (TCZ), as monotherapy. Although the significance of IL-6 as a conceptual target for cancer treatment is well-documented [8, 54, 65, 66], we still do not fully understand how anti-IL6 therapies work in vivo. Our simulations predict that as little as 1mg/kg of TCZ administered weekly for 7 weeks is sufficient to result in tumor reduction and a sustained tempering of tumor growth. The observed effect of TCZ is due to the fact that it competes with IL-6 for the signaling receptor, IL-6R. The model also predicts that administration of doses as small as 1mg/kg of TCZ is sufficient for saturating IL-6R with TCZ molecules. We expect that larger doses of TCZ may be necessary to achieve this same type of interference when xenografts include both human endothelial cells and human tumor cells, as in this case the amount of competing IL-6 in the tumor microenvironment will significantly increase.

We were interested to know if TCZ had greater impact on cell death or on cancer stem cell self-renewal, as changes in self-renewal and death rates of CSCs can alter the final proportion of CSCs post-treatment. The model predicts that the effect of TCZ on cell death is more pronounced than its effect on CSC self-renewal, and this leads to a small increase in the percentage of CSCs after treatment with TCZ. Therefore, for these primary tumor xenografts that do not include human endothelial cells, TCZ results in overall tumor reduction, but a final tumor composition that has a slightly larger proportion of CSCs. In [33], the effect of TCZ on Mucoepidermoid carcinoma (MEC) cell lines is investigated in an experimental setting that included human endothelial cells. In that study, they showed that TCZ has mixed effects on the fraction of CSCs post therapy. Interestingly, our preliminary model simulations of an experimental system that includes SCC cell lines and human endothelial cells shows that TCZ leads to a decrease in the proportion of cancer stem cells.

### Conclusion

It is clear that IL-6 plays a critical role in the pathobiology of cancer, due in part to its impact on cancer stem cells. This has provided strong rationale for developing targeted inhibitors of IL-6. This modeling study not only quantifies the influence on IL-6 on primary tumor xenografts; it also provides some explanations for the various effects of TCZ on tumor growth and CSC percentage. We are currently modifying this model to describe xenografts that include human endothelial cells that have been demonstrated to produce IL-6 in greater amounts than tumor cells. Preliminary results for this approach, some of which were described above, are promising. We will also extend the model to include combination therapies with traditional chemotherapeutic agents, like cisplatin. This extended model can be used to simulate different dose-scheduling regimens in order to investigate synergism between the two therapies. Continued modeling efforts in this direction have the potential to shed light on conditions under which TCZ sensitizes cancer cells for treatment with cisplatin and can be used to predict the optimal dose scheduling that will lead to maximal tumor response.

## Supporting information

S1 Appendix Full TCZ treatment model. (PDF)
