# Supplementary material for: A mathematical model for IL-6-mediated, stem cell driven tumor growth and targeted treatment
Source: PLoS Comput Biol. 2018 Jan 19;14(1):e1005920. doi: 10.1371/journal.pcbi.1005920 (PMC5792033; doi:10.1371/journal.pcbi.1005920)
Supplement: S1 Appendix — (PDF) [file pcbi.1005920.s001.pdf]

A mathematical model for IL-6-mediated, stem cell driven tumor growth and targeted treatment

Fereshteh Nazari Alexander T. Pearson Jacques Eduardo Nör Trachette L. Jackson

S1 APPENDIX

Variables related to the anti-IL-6R treatment model are provided in Table S1 below.

Table S1: Variables related to anti-IL-6R treatment model

Variable	Description	Units
S	HNSCC stem cells	# of cells
E	HNSCC progenitor tumor cells	# of cells
D	HNSCC differentiated tumor cells	# of cells
L	IL-6	fmol
R_S	IL-6R on stem cells	fmol
C_S	IL-6, cell bound IL-6R complex on stem cells	fmol
R_E	IL-6R on progenitor cells	fmol
C_E	IL-6, cell bound IL-6R complex on progenitor cells	fmol
R_D	IL-6R on differentiated cells	fmol
C_D	IL-6, cell bound IL-6R complex on differentiated cells	fmol
I	Free anti-IL-6R antibody in the tumor	fmol
C_S^I	Anti-IL-6R–cell bound IL-6R complex on stem cells	fmol
C_E^I	Anti-IL-6R–cell bound IL-6R complex on progenitor cells	fmol
C_D^I	Anti-IL-6R–cell bound IL-6R complex on differentiated cells	fmol

The model equations used for simulating tumor cells and drug pharmacokinetics for the anti-IL-6 antibody TCZ are described in detail in the main text. Below we present the full model equations associated with this targeted treatment.

TCZ-Therapy Model

$$\frac{dS}{dt} = \underbrace{\alpha_S P_S(S,\phi_S)S}_{\text{Stem cell self-renewal}} - \underbrace{\frac{\delta_S}{1+\gamma_S\phi_S}S}_{\text{Stem cell death}}$$

$$\frac{dE}{dt} = \underbrace{A_{in}\alpha_S(1-P_S(S,\phi_S))S}_{\substack{\text{Amplified}\\ \text{stem cell differentiation}}} - \underbrace{\alpha_E\, E}_{\substack{\text{Progenitor cell}\\ \text{differentiation}}} - \underbrace{\frac{\delta_E}{1+\gamma_E\phi_E}E}_{\text{progenitor cell death}}$$

$$\frac{dD}{dt} = \underbrace{A_{out}\alpha_E E}_{\substack{\text{progenitor cell}\\\text{differentiation}}} - \underbrace{\frac{\delta_D}{1+\gamma_D\phi_D} D}_{\text{differentiated cell death}}$$

$$\begin{aligned}\frac{dL}{dt} = & - \underbrace{k_f\, L\, R_S}_{\substack{\text{IL6 binding to}\\\text{stem cells}}} + \underbrace{k_r\, C_S}_{\substack{\text{IL6 dissociation}\\\text{from stem cells}}} - \underbrace{k_f\, L\, R_E}_{\substack{\text{IL6 binding to}\\\text{progenitor cells}}} + \underbrace{k_r\, C_E}_{\substack{\text{IL6 dissociation}\\\text{from progenitor cells}}}\\ & - \underbrace{k_f\, L\, R_D}_{\substack{\text{IL6 binding to}\\\text{differentiated cells}}} + \underbrace{k_r\, C_D}_{\substack{\text{IL6 dissociation}\\\text{from differentiated cells}}} - \underbrace{\lambda_L\, L}_{\text{IL6 natural decay}} + \underbrace{\rho(S+E+D)}_{\substack{\text{IL-6 Production}\\\text{by tumor cells}}}\end{aligned}$$

$$\begin{aligned}\frac{dI}{dt} = & - \underbrace{k_f^I\, I\, R_S}_{\substack{\text{Anti-IL6R binding to}\\\text{stem cells}}} + \underbrace{k_r^I\, C_S^I}_{\substack{\text{Anti-IL6R dissociation}\\\text{from stem cells}}} - \underbrace{k_f^I\, I\, R_E}_{\substack{\text{Anti-IL6R binding to}\\\text{progenitor cells}}} + \underbrace{k_r^I\, C_E^I}_{\substack{\text{Anti-IL6R dissociation}\\\text{from progenitor cells}}}\\ & - \underbrace{k_f^I\, I\, R_D}_{\substack{\text{Anti-IL6R binding to}\\\text{differentiated cells}}} + \underbrace{k_r^I\, C_D^I}_{\substack{\text{IL6 dissociation}\\\text{from differentiated cells}}} + \underbrace{k_{12} I_s - k_{21} I}_{\text{Pharmacokinetics}}\end{aligned}$$

$$\begin{aligned}\frac{dR_S}{dt} = & - \underbrace{k_f\, L\, R_S}_{\substack{\text{IL-6 binding to}\\\text{stem cells}}} + \underbrace{k_r\, C_S}_{\substack{\text{IL-6 dissociation}\\\text{from stem cells}}} + \underbrace{k_p C_S}_{\text{Recycling}} - \underbrace{k_f^I\, I\, R_S}_{\substack{\text{Anti-IL6R binding to}\\\text{IL-6R}}} + \underbrace{k_r^I\, C_S^I}_{\substack{\text{Anti-IL-6R dissociation}\\\text{from stem cells}}}\\ & + \underbrace{R_{T_S}\, \mathscr{P}_{\mathscr{S}}(S,\phi_S)}_{\substack{\text{Generation of new } R_S\\\text{via cell proliferation}}} - \underbrace{\frac{R_S}{R_S + C_S + C_S^I} R_{T_S}\, \mathscr{D}_{\mathscr{S}}(S,\phi_S)}_{\substack{\text{Loss of } R_S\\\text{via cell death}}}\end{aligned}$$

$$\frac{dC_S^I}{dt} = + \underbrace{k_f^I\, I\, R_S}_{\substack{\text{Anti-IL6R binding to}\\\text{IL-6R}}} - \underbrace{k_r^I\, C_S^I}_{\substack{\text{Anti-IL6R dissociation}\\\text{from stem cells}}} - \underbrace{\frac{C_S^I}{R_S + C_S + C_S^I} R_{T_S}\, \mathscr{D}_{\mathscr{S}}(S,\phi_S)}_{\substack{\text{Loss of } C_S^I\\\text{via cell death}}}$$

$$\frac{dC_S}{dt} = + \underbrace{k_f\, L\, R_S}_{\text{IL6 binding to } R_S} - \underbrace{k_r\, C_S}_{\substack{\text{IL6 dissociation}\\\text{from } R_S}} - \underbrace{k_p C_S}_{\text{Internalization}} - \underbrace{\frac{C_S}{R_S + C_S + C_S^I} R_{T_S}\, \mathscr{D}_{\mathscr{S}}(S,\phi_S)}_{\substack{\text{Loss of } C_S\\\text{via cell death}}}$$

$$\begin{aligned}\frac{dR_E}{dt} = & - \underbrace{k_f\, L\, R_E}_{\substack{\text{IL-6 binding to}\\\text{progenitor cells}}} + \underbrace{k_r\, C_E}_{\substack{\text{IL-6 dissociation}\\\text{from progenitor cells}}} + \underbrace{k_p C_E}_{\text{Recycling}} - \underbrace{k_f^I\, I\, R_E}_{\substack{\text{Anti-IL6R binding to}\\\text{IL-6R}}} + \underbrace{k_r^I\, C_E^I}_{\substack{\text{Anti-IL-6R dissociation}\\\text{from progenitor cells}}}\\ & + \underbrace{R_{T_E}\, \mathscr{P}_{\mathscr{E}}(E,\phi_E)}_{\substack{\text{Generation of new } R_E\\\text{via cell proliferation}}} - \underbrace{\frac{R_E}{R_E + C_E + C_E^I} R_{T_E}\, \mathscr{D}_{\mathscr{E}}(E,\phi_E)}_{\substack{\text{Loss of } R_E\\\text{via cell death}}}\end{aligned}$$

$$\frac{dC_E^I}{dt} = + \underbrace{k_f^I\, I\, R_E}_{\substack{\text{Anti-IL6R binding to}\\\text{IL-6R}}} - \underbrace{k_r^I\, C_E^I}_{\substack{\text{Anti-IL6R dissociation}\\\text{from progenitor cells}}} - \underbrace{\frac{C_E^I}{R_E + C_E + C_E^I} R_{T_E}\, \mathscr{D}_{\mathscr{E}}(E,\phi_E)}_{\substack{\text{Loss of } C_E^I\\\text{via cell death}}}$$

$$\frac{dC_E}{dt} = + \underbrace{k_f\, L\, R_E}_{\text{IL6 binding to } R_E} - \underbrace{k_r\, C_E}_{\substack{\text{IL6 dissociation} \\ \text{from } R_E}} - \underbrace{k_p C_E}_{\text{Internalization}} - \underbrace{\frac{C_E}{R_E + C_E + C_E^I} R_{T_E}\, \mathscr{D}_{\mathscr{E}}(E, \phi_E)}_{\substack{\text{Loss of } C_E \\ \text{via cell death}}}$$

$$\frac{dR_D}{dt} = - \underbrace{k_f\, L\, R_D}_{\substack{\text{IL-6 binding to} \\ \text{differentiated cells}}} + \underbrace{k_r\, C_D}_{\substack{\text{IL-6 dissociation} \\ \text{from differentiated cells}}} + \underbrace{k_p C_D}_{\text{Recycling}} - \underbrace{k_f^I\, I\, R_D}_{\substack{\text{Anti-IL6R binding to} \\ \text{IL-6R}}} + \underbrace{k_r^I\, C_D^I}_{\substack{\text{Anti-IL-6R dissociation} \\ \text{from differentiated cells}}}$$
$$+ \underbrace{R_{T_D}\, \mathscr{P}_{\mathscr{D}}(E, \phi_D)}_{\substack{\text{Generation of new } R_D \\ \text{via cell proliferation}}} - \underbrace{\frac{R_D}{R_D + C_D + C_D^I} R_{T_D}\, \mathscr{D}_{\mathscr{D}}(E, \phi_D)}_{\substack{\text{Loss of } R_D \\ \text{via cell death}}}$$

$$\frac{dC_D^I}{dt} = + \underbrace{k_f^I\, I\, R_D}_{\substack{\text{Anti-IL6R binding to} \\ \text{IL-6R}}} - \underbrace{k_r^I\, C_D^I}_{\substack{\text{Anti-IL6R dissociation} \\ \text{from differentiated cells}}} - \underbrace{\frac{C_D^I}{R_D + C_D + C_D^I} R_{T_D}\, \mathscr{D}_{\mathscr{D}}(D, \phi_D)}_{\substack{\text{Loss of } C_D^I \\ \text{via cell death}}}$$

$$\frac{dC_D}{dt} = + \underbrace{k_f\, L\, R_D}_{\text{IL6 binding to } R_D} - \underbrace{k_r\, C_D}_{\substack{\text{IL6 dissociation} \\ \text{from } R_D}} - \underbrace{k_p C_D}_{\text{Internalization}} - \underbrace{\frac{C_D}{R_D + C_D + C_D^I} R_{T_D}\, \mathscr{D}_{\mathscr{D}}(D, \phi_D)}_{\substack{\text{Loss of } C_D \\ \text{via cell death}}}$$

It is worth mentioning that similar to our calculations for the pretreatment model, it can be shown that the equations for treatment satisfy:

$$\begin{aligned} R_S &= R_{T_S} S - C_S - C_S^I \\ R_E &= R_{T_E} E - C_E - C_E^I \\ R_D &= R_{T_D} D - C_D - C_D^I \end{aligned}$$

This model is fully parameterized in the main text, with the exception of the TCZ association and dissociation rates, which are provided in Table S2 below.

Table S2: TCZ Binding Parameters

**Parameters**	**Values**	**Units**	**Reference**
k_f^I	$8.5/\mathrm{Vol}_T$*	$\mathrm{fmol}^{-1}\mathrm{day}^{-1}$	[1]
k_r^I	21.6	day^{-1}	lens.org/092-218-642-694-833

*Vol_T is the volume of the tumor in μl and is equal to (volume of 1 million tumor cells) $\times$ (S+E+D), where, the volume of 1 tumor cell is $1 \times 10^{-6} \mu l$ [2].

Notes on Normalizing the Stem Cell Percentage

We were unable to obtain information on the percentage of CSCs in the primary tumors used in the experiments shown in Figure 13. However, from the literature, we found that stem cells in

primary HNSCC tumors seem to vary between 0.6 and 3.2% [3, 4, 5, 6]. Therefore, we chose 2.1%, which is in the range we found in the literature, as the baseline CSC percentage in human primary tumors and then normalized it to 1%. This means that, in the first bar the 1% in the Figure 13 corresponds to 2.1% in the primary tumor after surgery, and in the second bar, 1.2% corresponds to 2.52% CSCs in the xenograft tumor at day 121.

Furthermore, numerical analysis of our model suggest that choosing any value between 0.6 and 3.2 as the initial percentage of the CSCs in primary human tumor does not quantitatively change the growth dynamics of xenograft tumors.

References

1. Mihara M, Kasutani K, Okazaki M, Nakamura A, Kawai S, Sugimoto M, et al. Tocilizumab inhibits signal transduction mediated by both mIL-6R and sIL-6R, but not by the receptors of other members of IL-6 cytokine family. International immunopharmacology. 2005;5(12):1731–1740.

2. Cunningham SA, Tran TM, Arrate MP, Brock TA. Characterization of Vascular Endothelial Cell Growth Factor Interactions with the Kinase Insert Domain-containing Receptor Tyrosine Kinase A Real Time Kinetic Study. Journal of Biological Chemistry. 1999;274(26):18421–18427.

3. Bao B, Ahmad A, Azmi AS, Ali S, Sarkar FH. Chapter 14. In: Enna SJ, editor. Overview of cancer stem cells (CSCs) and mechanisms of their regulation: Implications for cancer therapy. Wiley Online Library; 2013. p. 14–25.

4. Clay MR, Tabor M, Owen JH, Carey TE, Bradford CR, Wolf GT, et al. Single-marker identification of head and neck squamous cell carcinoma cancer stem cells with aldehyde dehydrogenase. Head & neck. 2010;32(9):1195–1201.

5. Reid P, Wilson P, Li Y, Marcu LG, Staudacher AH, Brown MP, et al. In vitro investigation of head and neck cancer stem cell proportions and their changes following X-ray irradiation as a function of HPV status. PLoS One. 2017;12(10):e0186186.

6. Shrivastava S, Steele R, Sowadski M, Crawford SE, Varvares M, Ray RB. Identification of molecular signature of head and neck cancer stem-like cells. Scientific Reports. 2015;5:7819.
